# Parallel G-Quadruplex DNA Structures from Nuclear and Mitochondrial Genomes Trigger Emission Enhancement in a Nonfluorescent Nano-aggregated Fluorine–Boron-Based Dye

**DOI:** 10.1021/acs.jpclett.2c03301

**Published:** 2023-02-13

**Authors:** Marco Deiana, Karam Chand, Erik Chorell, Nasim Sabouri

**Affiliations:** † Department of Medical Biochemistry and Biophysics, Umeå University, 90187 Umeå, Sweden; ‡ Department of Chemistry, Umeå University, 90187 Umeå, Sweden

## Abstract

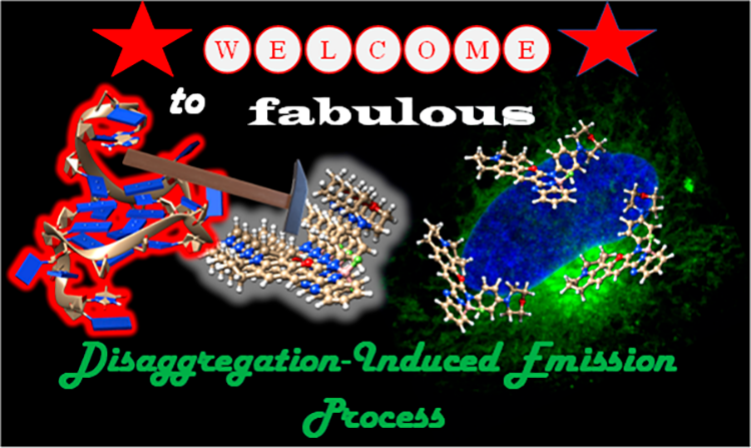

Molecular self-assembly is a powerful tool for the development of functional nanostructures with adaptive optical properties. However, in aqueous solution, the hydrophobic effects in the monomeric units often afford supramolecular architectures with typical side-by-side π-stacking arrangement with compromised emissive properties. Here, we report on the role of parallel DNA guanine quadruplexes (G4s) as supramolecular disaggregating–capture systems capable of coordinating a zwitterionic fluorine–boron-based dye and promoting activation of its fluorescence signal. The dye’s high binding affinity for parallel G4s compared to nonparallel topologies leads to a selective disassembly of the dye’s supramolecular state upon contact with parallel G4s. This results in a strong and selective disaggregation-induced emission that signals the presence of parallel G4s observable by the naked eye and inside cells. The molecular recognition strategy reported here will be useful for a multitude of affinity-based applications with potential in sensing and imaging systems.

The nature of Hoogsteen base pairing in DNA enables control over the folding of higher-order structures featuring a number of different molecular architectures.^1^ In particular, noncanonical four-stranded DNA structures such as intercalated motifs (i-DNAs) and G4s have received increasing scientific attention, not only for being implicated in key biological processes,^2−4^ but also for their predictable and controllable properties. Indeed, such structures can afford complex nanometer scale systems suited for molecular computing,^5,6^ transport,^7,8^ amplification of chirality,^9^ and biosensing^10−17^ applications. While i-DNAs are predominantly formed under acidic conditions (pH < 7),^18−20^ G4s are thermodynamically stable at physiological pH.^21,22^ G4s are composed of stacked G-tetrads octa-coordinated by a central metal ion (usually K^+^) and further stabilized by intraquartet hydrogen bonds.^23,24^ Owing to their polynucleotide constitution and dynamic conformation, G4s show high polymorphism in solution. Based on their structural features, G4s are commonly classified into different topologies, namely, parallel, hybrid, or antiparallel, and the development of small-molecule probes selective for certain G4 classes still remains one of the biggest challenges in the field for its vast biomedical potential.^23,24^

The development of luminescent materials coupled with the rapid evolution of optical microscopy techniques has allowed generation of valuable knowledge in the roles and functions that G4s play in cells.^10,11,25^ However, in many conventional systems, fluorophores experience the phenomenon of aggregation-induced quenching (ACQ) that limits their full potential as diagnostic agents.^26^ Recently, we^27−29^ and others^30−35^ have introduced a selective G4 recognition strategy that relies on the disassembly of self-aggregated dyes, namely, disaggregation-induced emission (DIE). Dyes operating through DIE display remarkable topological/sequence selectivity^27−29,33−35^ for G4s and have broad applicability in cellular systems.^27,30,35^

The coumarin core can be combined with, for example, quinazolines, benzothiazoles, benzoimidazoles, and amidines to give compounds that combine fluorescent properties with G4 binding and stabilizing properties.^12,17^ Along the same line, dipyrro-boradiazaindacenes (BODIPY) dyes have great potential in the field of fluorescent sensing and bioimaging because of their excellent photophysical properties, relatively high photostability, and neutral total charge.^36,37^ Unfortunately, with some exceptions,^38−40^ the strong intermolecular interactions exerted by the planar π-conjugated BODIPY-units, in aqueous solution, strongly quench the fluorescence intensity through activation of fast nonradiative decay pathways, narrowing their practical applications.^41^ Here, by building on this knowledge, we designed a benzimidazole–iminocoumarin fused with a fluorine–boron unit that resembles the BODIPY core to generate a self-assembled dye, hereafter **4b**, capable of recovering its intense fluorescence in response to binding to parallel G4s through the recognition-induced disassembly of the nonemissive aggregates (Figure 1). The associated binary OFF–ON fluorescence output makes it possible to track the molecular distribution of **4b** in live cells. This shows accumulation of the probe in the mitochondrial networks which are known to contain hundreds of potential DNA G4-forming sequences, with many forming parallel G4 structures (Figure 1).^42,43^

**Figure 1:**
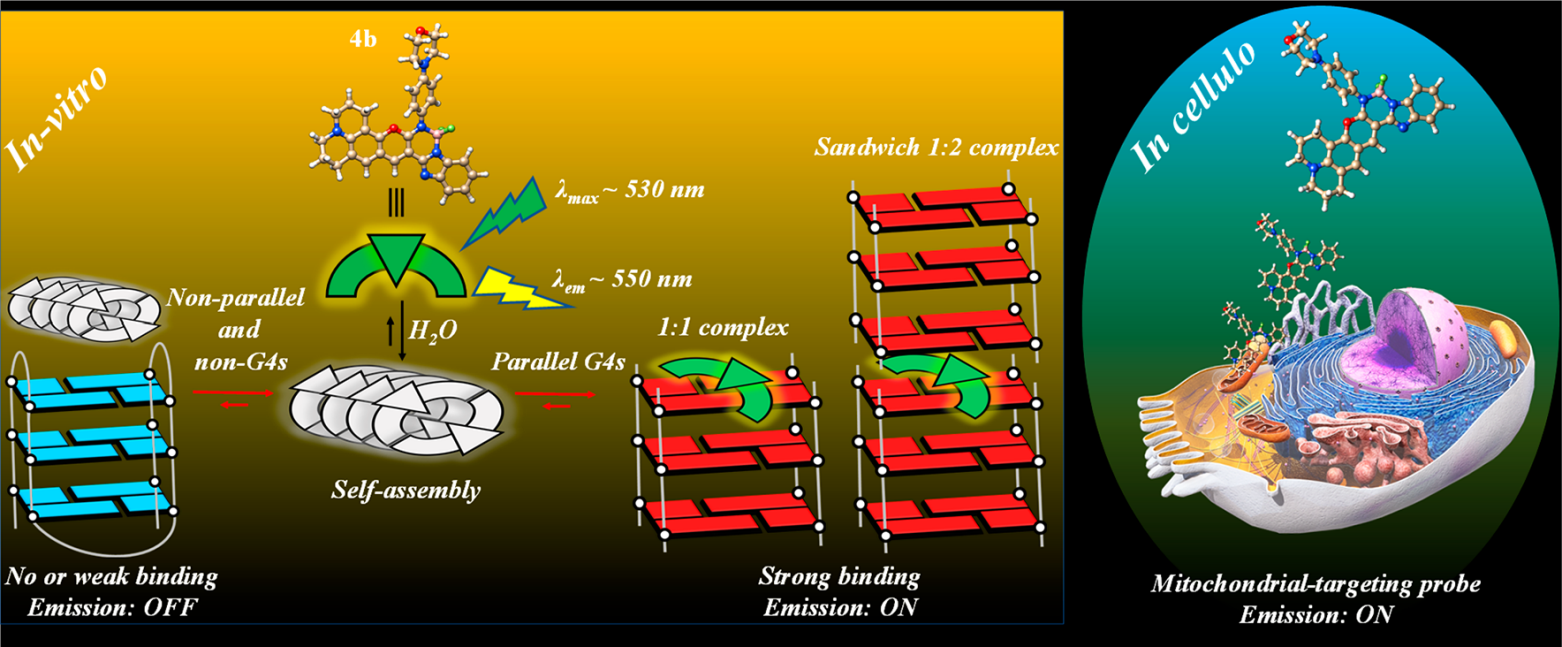
Schematic representation deciphering the DIE process. **4b** forms nanoaggregates with a self-quenched emission profile and inherently low background signal. Coordination with parallel G4s drives the disassembly of the **4b** aggregates into stable and highly emissive 1:1 or 1:2 sandwiched parallel G4-**4b** complexes. On the other hand, nonparallel and non-G4s have almost no effect on **4b**’s intensiometric response. In cancer cells, **4b** accumulates within the mitochondrial networks which are known to host hundreds of potential parallel G4-forming sequences.

To generate the self-assembled dyes with adaptive and responsive G4-binding properties, we designed and synthesized two benzimidazole–iminocoumarins based on fluorine–boron units in three steps (Scheme 1 and Figures S1–S5). In the first step, commercially available 8-hydroxyjulolidine-9-carboxaldehyde (**1**) was subjected to Knoevenagel condensation with 2-benzimidazoleacetonitrile in the presence of catalytic amounts of piperidine in anhydrous ethanol to form the benzimidazole–iminocoumarin (**2**) intermediate in 66% yield. In the second step, transamination reaction of **2** was performed with the appropriate anilines in the presence of catalytic amounts of tosylic acid to give the desired benzimidazole–iminocoumarins (**3a**,**b)** in good yields (69%–78%). The difluoroboron(III) units were finally installed by using boron trifluoride diethyl etherate (BF_3_–OEt_2_) in refluxing dichloroethane in the presence of base to give the desired dyes (**4a**,**b**) in 58%–73% yield.

**Scheme 1:**
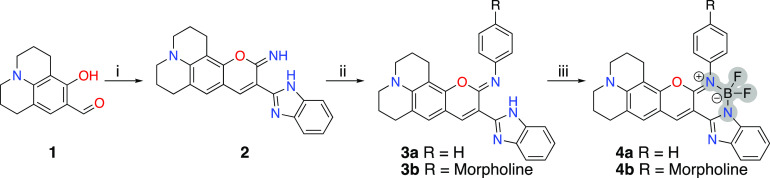
Synthetic Route Reagents and conditions: (i) cat. piperidine, 2-benzimidazoleacetonitrile, absolute ethanol, RT, 12 h; (ii) aniline/4-morpholinoaniline, cat. P-TsOH, ethanol, reflux 12–14 h; (iii) BF_3_-OEt_2_, DIPEA, reflux, 2–3 h.

**4a** and **4b** revealed fairly good solubility in various organic solvents and a moderate overall positive solvatochromism as evidenced by absorption and steady-state emission studies (Figure S6). In aqueous solution, **4a** and **4b** absorption spectra showed relatively broad and weakly structured transition bands with apparent extinction coefficient *ε*_app_ ≈ 2.0 × 10^4^ M^–1^ cm^–1^ and *ε*_app_ ≈ 2.4 × 10^4^ M^–1^ cm^–1^ at 495 and 499 nm, respectively (Figures 2A and S7A). These spectral features hinted at the potential ability of the compounds to form intermolecular H-type aggregates with side-by-side stacked structures.^27,29^ Indeed, dynamic light scattering (DLS) and atomic force microscopy (AFM) revealed the presence of small nanometer-sized particles with an average size of ∼160 nm supporting the formation of nanoaggregates (Figures 2A, S7A, and S8). To test the switchable assembly–disassembly behavior of **4a** and **4b**, we first performed UV/vis titration studies by using sodium dodecyl sulfate (SDS), which is known to be a very efficient disaggregating agent.^27,29^ At a concentration below 1.5 × 10^–3^ M, the surfactant did not affect the formation of the aggregated molecules (Figures 2A and S7A). Conversely, above the critical concentration of ∼1.6 × 10^–3^ M, SDS induced a marked breakup of the compounds’ aggregates that resulted in the formation of well-defined and bathochromically shifted transition bands centered at ∼520 nm which are ascribed to the monomeric state of the compounds (Figures 2A and S7A). Steady-state emission measurements unraveled the presence of self-quenched emissive bands supporting the presence of radiationless relaxation pathways available in side-by-side stacked-structures with H-type character (Figures 2B and S7B).^26^ On the other hand, the gradual addition of SDS turned on the emissive properties of the compounds, providing a bright fluorescent band centered at ∼545 nm (Figures 2B and S7B). The excitation spectra measured for both the compounds at their monomer-like emission band well-represented the associated absorption bands, supporting a reversible and efficient SDS-induced disaggregation process (Figure S9).

**Figure 2:**
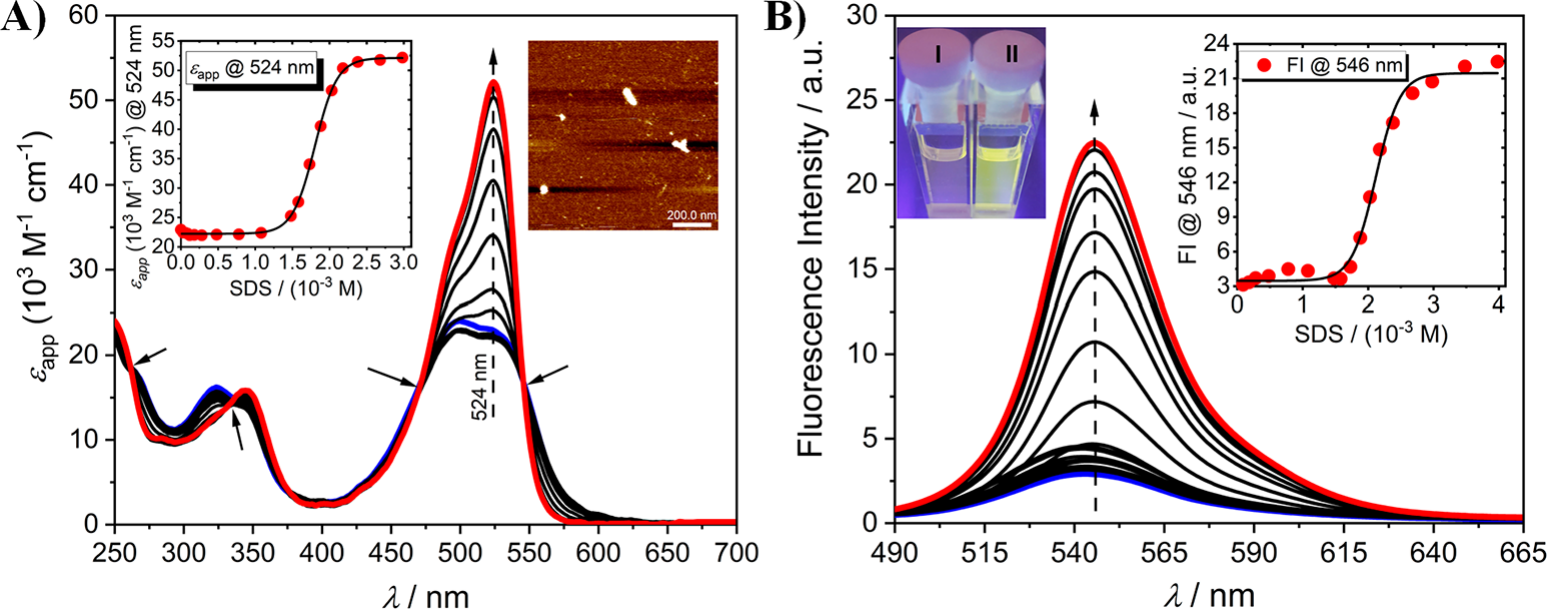
UV/vis absorption (A) and steady-state emission (B) spectra of **4b** (*c*_**4b**_ = 3 μM, c_TRIS_ = 50 mM, pH 7.4, blue line, *λ*_exc_ = 470 nm) with increasing concentrations of SDS (*c*_SDS_ = 0 to 2.98 mM in panel A and 0 to 3.98 mM in panel B). The insets in panel A show the spectral evolution profile of **4b** at 524 nm in the presence of SDS and a representative AFM height image of **4b**-nanoaggregates (*c*_**4b**_ = 10 μM, *c*_TRIS_ = 10 mM, pH 7.4). The insets in panel B show the spectral evolution profile of **4b** at 546 nm in the presence of SDS and the resulting SDS-induced color change on **4b**.

The controlled self-assembly process coupled with the dramatic changes in the collective optical response of the molecules prompted us to investigate the properties of **4a** and **4b** in the presence of DNA G4s, which are well-known targets in anticancer drug development.^44^ For these investigations, we selected a panel of biologically relevant and well-characterized G4s with various topologies: parallel, hybrid, and antiparallel (Table S1). Binding of **4b** to a parallel G4 (*c-KIT* 2) derived from the promoter region of the *c-KIT* oncogene led to significant absorption spectral changes reminiscent of the disassembly observed with the surfactant (Figures 2A, 3A, and S10). Indeed, an intense structured absorption band centered at ∼530 nm along with the appearance of well-defined isosbestic points at ∼513 and 550 nm clearly point toward the formation of stable complexes between **4b** and this parallel G4 DNA. In contrast, addition of **4a** to the *c-KIT* 2 G4 or other parallel and hybrid G4s showed less pronounced absorption spectral changes, probably due to its diminished G4-binding properties that hamper the disassembly process (Figure S11).

**Figure 3:**
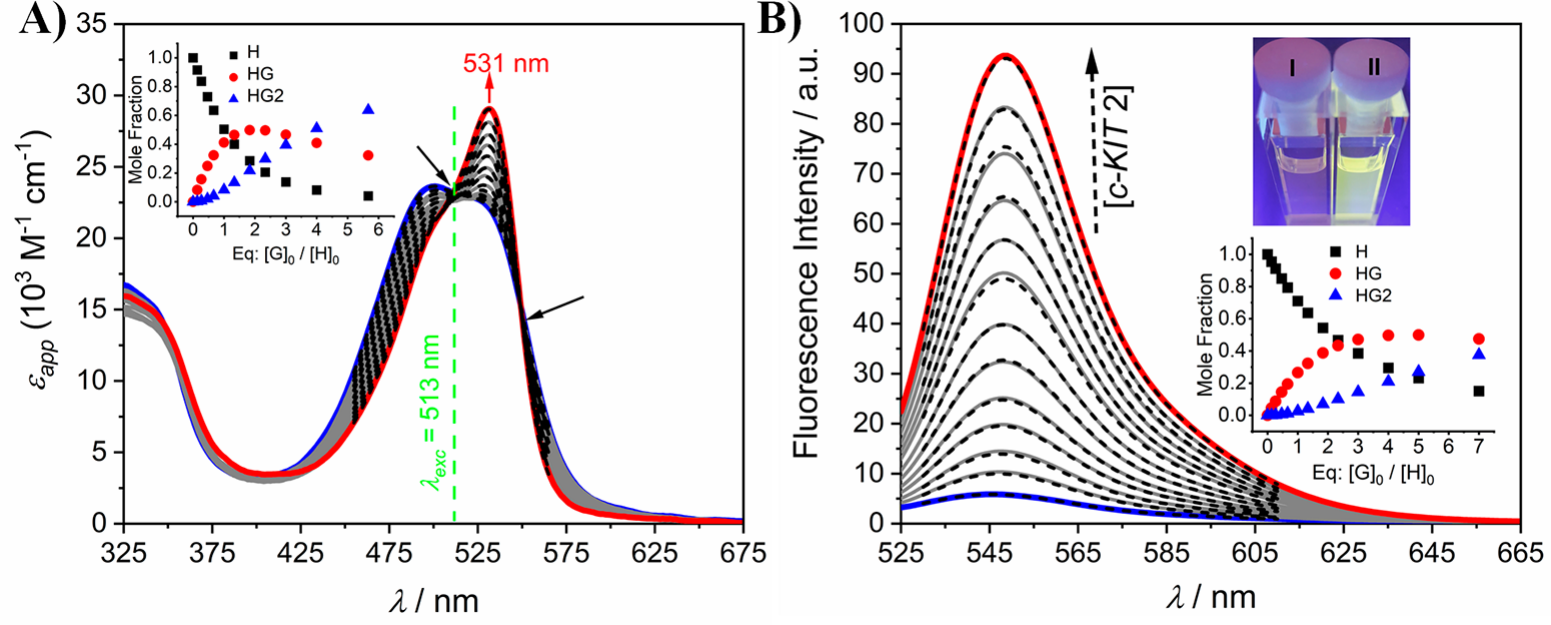
(A) UV/vis absorption spectra of **4b** in the absence (blue line) and presence (gray and red lines) of increasing concentrations of *c-KIT* 2 (*c*_**4b**_ = 3 μM, *c*_*c-KIT* 2_ = 0 to 17 μM, *c*_KCl_ = 100 mM, *c*_TRIS_ = 10 mM, pH 7.4). Superimposed black dashed line results from a global fitting with a 1:2 (K_12_) binding model. The solid arrows indicate the appearance of isosbestic points, and the green vertical line aims to show the excitation wavelength used to record the emission spectra in order to avoid changes in the optical density. The inset shows the speciation analysis for the titration of **4b** with *c-KIT* 2 derived using the values of K_12_ obtained by fitting as described by Thordarson and co-workers.^45,46^ [G]_0_ = free *c-KIT* 2 concentration, [H]_0_ = free **4b** concentration. H, HG, and HG2 are the components present in the system and indicate **4b**, **4b**-(*c-KIT* 2) 1:1 adduct, and **4b**-(*c-KIT* 2)2 1:2 adduct, respectively. (B) Steady-state emission spectra of **4b** in the absence (blue line) and presence (gray and red lines) of increasing concentrations of *c-KIT* 2 (*c*_**4b**_ = 3 μM, *c*_*c-KIT* 2_ = 0 to 21 μM, *c*_KCl_ = 100 mM, *c*_TRIS_ = 10 mM, pH 7.4, *λ*_exc_ = 513 nm). Superimposed black dashed lines result from a global fitting with a 1:2 binding model. The insets show the evolution of the speciation analysis as in panel A and the resulting color change of **4b** raised from the complexation with *c-KIT* 2 (I = **4b** alone, II = **4b**:*c-KIT* 2 complex).

Since the only difference between the two compounds lies in the presence of an additional morpholine group located on **4b**’s scaffold, we believe that this substituent may play pivotal roles in providing additional G4-interactive binding properties to the molecule by acting as proton acceptor and participating in hydrogen bonding interactions. Indeed, we recently showed that a quinazoline–quinazolinone bearing a morpholine group had the strongest G4-stabilizing effect among closely related analogues.^47^ In light of these results, we thus decided to focus our full attention on **4b**. In line with the G4-induced absorption spectral changes, steady-state emission studies showed a dramatic increase in the fluorescence intensity of **4b** bound to parallel G4s which is visible to the naked eye (Figures 3B and S12). Conversely, no or very weak binding response could be detected for **4b** complexed with hybrid, antiparallel, and non-G4 forming sequences (single-stranded (ss) and double-stranded (ds) DNAs) indicating a clear-cut binding preference of **4b** for parallel G4s (Figures S13 and S14). The ability of **4b** to discriminate nonparallel G4 motifs is a rather unique feature of this dye since previously reported self-assembled molecules exhibited off-target binding to nonparallel and non-G4 structures.^32,35^ Moreover, **4b**’s emissive properties were almost unaltered by changes in pH, as well as by the presence of various ions, metabolites, and extracellular plasma proteins such as bovine serum albumin (BSA) (Figure S15). For instance, the addition of two equivalents of BSA barely increased **4b**’s emission to 2-fold (Figure S15), compared to about 8-fold increase in the presence of a parallel G4 DNA (Figure 4B). Next, detailed quantitative binding data analysis^45,46^ was performed on both absorption and emission spectra that provided association constants (K or K_12_) in the order of 10^5^ M^–1^ with binding stoichiometries that differ based on the type of G4 sequence used (Figure 4A,B and Table S2). For instance, the use of the 1:2 binding model turned out to be the most accurate fitting procedure for **4b** complexed with *c-KIT* 2, *c-MYC* Pu22, *VEGF*, *BCL*-2, and *c-KIT* 87up G4s (Figures S16 and S17). The tangent method analysis for the **4b**-*c-KIT* 2 complex further supported this binding mode (Figure S18). This sandwich-type complex features **4b** intercalated between two terminal G-tetrad ends (Figure 1). A similar coordination process was previously reported for self-assembled squaraine dyes^29,31^ and for a platinum complex.^48^ No quantitative data analysis was performed for **4b** bound to nonparallel G4s as the saturation profile was not reached even upon the addition of a large excess of oligonucleotides. Time-dependent steady-state fluorescence studies, directed to determine the kinetic profile of **4b** complexed with various concentrations of *c-KIT* 2, clearly showed the ability of the G4 to catalyze the disassembly of **4b** nanoaggregates in a concentration-dependent manner (Figure S19).

**Figure 4:**
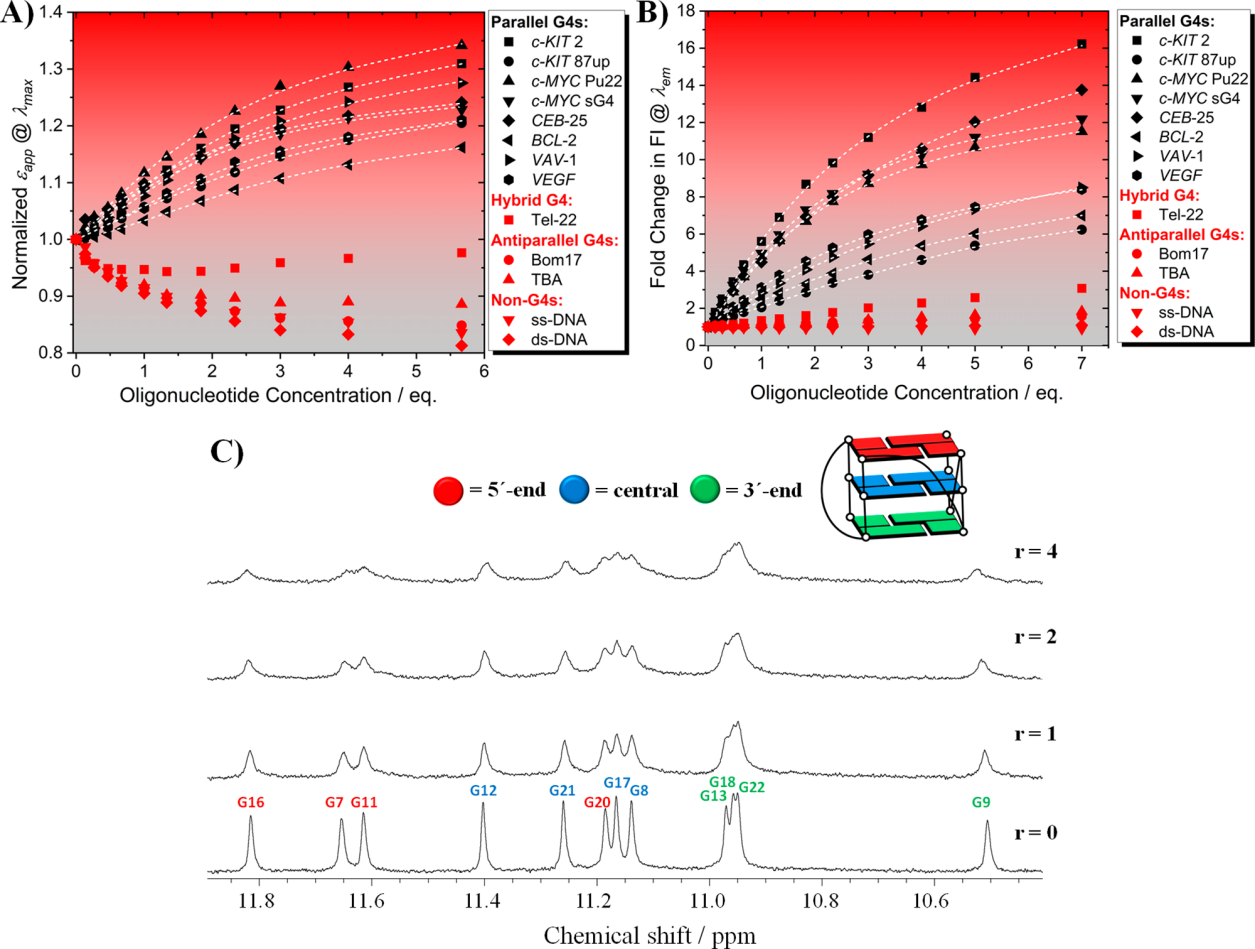
Spectrometric (A) and fluorimetric (B) titrations of **4b** with the G4 and non-G4 sequences given in the legend and described in Table S1 (*c*_**4b**_ = 3 μM, *c*_DNA sequences_ = 0 to 17 or 21 μM, *c*_KCl_ = 100 mM, *c*_TRIS_ = 50 mM, pH 7.4, *λ*_exc_ = 513 nm). The dashed white lines result from the fitting procedure displayed at *λ*_max_ or *λ*_em_ and summarized in Table S2. (C) Imino-proton regions of the 1D ^1^H NMR titration spectra of *c-MYC* Pu22 interacting with **4b** (*c*_**4b**_ = 0 to 400 μM, *c*_*c-MYC* Pu22_ = 100 μM, *c*_KCl_ = 50 mM, *c*_TRIS_ = 10 mM, pH 7.4, *T* = 25 °C). Ratios of **4b**/*c-MYC* Pu22 are shown in the spectra. The imino-proton assignments are labeled and color-coded based on their localization on the G4 template.

The interaction of **4b** and *c-KIT* 2 was then investigated by electronic circular dichroism (ECD) spectroscopy. CD data showed that the binding of **4b** to *c-KIT* 2 did not perturb its folding dynamic (Figure S20). To gain structural information about the **4b**–G4 coordination processes, we performed fluorescence displacement assays using two well-known G4-end stackers, Phen-DC_3_^49^ and BRACO-19^50^ (Figure S21). As expected, both Phen-DC_3_ and BRACO-19 were able to fully displace **4b** from the G-tetrad ends, highlighting the ability of **4b** to target the G4 template via an end-stacking binding mode. In order to determine the site localization of our probe, we performed 1D ^1^H NMR titration studies using the well-characterized *c-MYC* Pu22 G4 sequence (Figures 4C).^51^ We detected a clear line-broadening of the signals in the imino-region of *c-MYC* Pu22 compared to the DMSO-*d*_6_ control sample supporting the strong binding of the probe (Figure S22). These results were found to be in line with previous studies investigating the interaction of small-molecule ligands and G4s.^13,28,47^

Having established multiple details concerning the complexation process between **4b** and G4s *in vitro*, we then evaluated the cytotoxic effect of the probe in cancer cells over a 48 h treatment at various **4b** concentrations. **4b** did not elicit any significant cell death response even at 25 μM concentration, supporting its potential use as a noninvasive G4 probe (Figure 5A). Next, we evaluated the cellular emission fingerprint of **4b** by means of confocal laser scanning microscopy (CLSM). Live HeLa cells treated for 30 min with **4b** showed accumulation of the probe within the mitochondrial networks. In fact, the **4b** signal partially colocalized with MitoRed, a probe that localizes primary to the mitochondria (Figure 5B). However, we cannot exclude that **4b**, in addition to targeting the mitochondria, can also accumulate in the endoplasmic reticulum being part of its fluorescence signal confined within the nuclear lamina and perinuclear space. Mitochondria contain their own genetic material named mitochondrial DNA (mtDNA), a double-stranded circular molecule with a size of ∼17 kilobases.^52^ As mitochondria-targeting agents are a much sought-after class of compounds with great potential in both therapeutic and diagnostic applications, we asked if **4b** can bind G4 DNA sequences originated from mtDNA.^53,54^ Bioinformatic analysis predicted that the human mitochondrial genome harbors ∼200 putative G4-forming sequences with a density of potential G4s per kilobase that exceeds that of nuclear DNA,^42,43^ and sequences that can form G4s are associated with mtDNA deletion sites, supporting their formation in cells. We tested the ability of **4b** to light-up a panel of mtDNA G4 oligonucleotides that fold into different G4 topologies (Table S3).^42^ In fact, these emission studies clearly pointed out the ability of **4b** to recognize parallel G4s with strong discriminatory ability against other G4 topologies (Figure 5C). It is also important to note that **4b**’s signal was not observed in the nuclei of live HeLa cells, which may be a consequence of poor nuclear membrane permeability. To test this hypothesis, we fixed and permeabilized HeLa cells before treating them with **4b**. We detected the accumulation of **4b** at both extranuclear and nuclear levels with clear peaks in the subnuclear compartments whose appearance was compatible with that of nucleoli.^27,28,47^ Detection of nucleolar localization is typical for many fluorescent G4 ligands as nucleoli accommodate the multicopy G4-rich ribosomal genes.^12,13,27,28,47,16^ To validate the ability of **4b** to target G4 DNA structures in HeLa cells, we performed a competition assay by using the G4-specific ligand BRACO-19, which has been previously used to identify selective molecular probes for nuclear and mitochondrial DNA G4s.^56^ The **4b**-associated emission in the extranuclear and nuclear regions decreased significantly in the presence of increasing concentrations of BRACO-19, confirming the specific binding of **4b** to mitochondrial and nuclear DNA G4s (Figure 5D).

**Figure 5:**
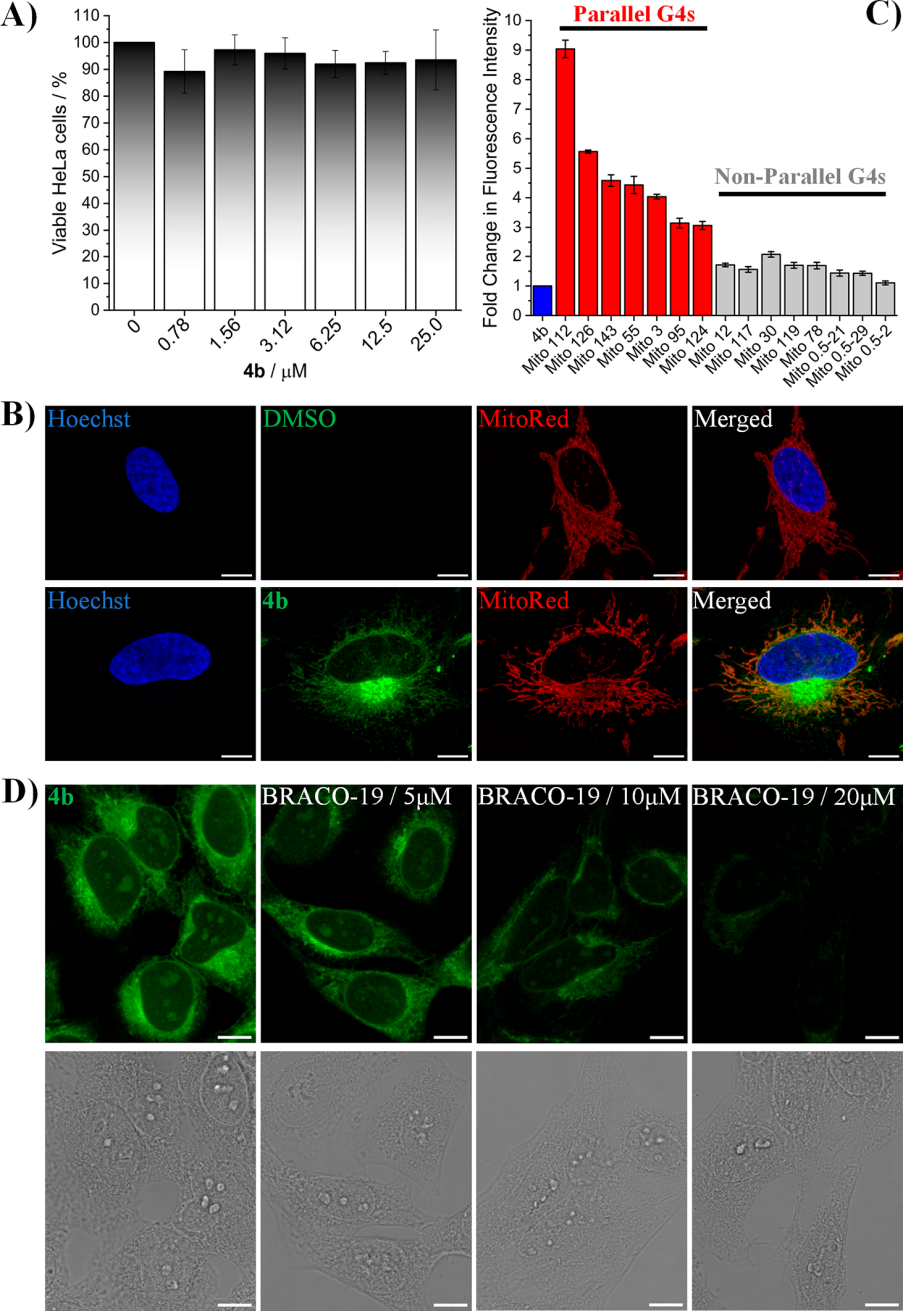
(A) Cytotoxic effects of **4b** on HeLa cells treated for 48 h. *N* = 4 (mean ± SD). (B) Live-cell imaging of HeLa cells treated with **4b** and incubated for 30 min (5 μM, green signal). HeLa cells were co-stained with the nuclear dye Hoechst 33342 (500 nM, blue signal) and MitoRed (100 nM, red signal). *λ*_exc_/*λ*_em_: 405/420–480 nm for Hoechst; 480/495–560 nm for **4b**; 580/590–715 for MitoRed. Scale bar 10 μm. (C) Fluorescence emission change of **4b** in the presence of mtDNA G4 sequences (*c*_**4b**_ = 1 μM, *c*_mtDNA G4 sequences_ = 2 μM, *c*_KCl_ = 100 mM, *c*_TRIS_ = 50 mM, pH 7.4, *λ*_exc_ = 513 nm). (D) Confocal (top) and bright field (bottom) images of MeOH-fixed HeLa cells treated with **4b** alone (5 μM, green signal) or incubated with different concentrations of BRACO-19 (5, 10, or 20 μM). Scale bar 10 μm.

To summarize, G4s are reported to play regulatory roles during mitochondrial transcription; however, unlike nuclear DNA G4s, still very little is known about other roles and functions of mtDNA G4s.^57−59^ Therefore, **4b** and its analogues may serve as an important noninvasive tool for better understanding of the functions of mtDNA G4s in live cells.

In summary, we have designed and synthesized a responsive self-assembled fluorine-boron-based dye-based dye capable of targeting and detecting parallel G4 structures. The disassembly of **4b**’s nanostructure is tightly regulated by strong directional noncovalent interactions and shape complementarity established exclusively with parallel G4s. Indeed, while parallel G4s triggered the formation of highly emissive sandwich-like complexes in which **4b** is intercalated between the two G-tetrad ends, nonparallel G4s have no significant effects on **4b**’s optical properties. Finally, the resulting binary OFF–ON fluorescent states enabled visualizing the mitochondrial networks in live HeLa cells which host hundreds of potential parallel G4-forming sequences. These findings open up new directions for adaptive nano systems involving G4 structures as optical modulators.

## Supporting Information Available

Synthesis and characterization of the compounds, experimental procedures, optical studies, self-assembly properties, G4-interactive binding studies, and fitting binding models (PDF)
