# Supplementary material for: Parallel G-Quadruplex DNA Structures from Nuclear and Mitochondrial Genomes Trigger Emission Enhancement in a Nonfluorescent Nano-aggregated Fluorine–Boron-Based Dye
Source: J Phys Chem Lett. 2023 Feb 13;14(7):1862–9. doi: 10.1021/acs.jpclett.2c03301 (PMC9940295; doi:10.1021/acs.jpclett.2c03301)
Supplement: Supplementary file 1 — jz2c03301_si_001.pdf [file jz2c03301_si_001.pdf]

Supporting Information

Parallel G-Quadruplex DNA Structures from Nuclear and Mitochondrial Genomes Trigger Emission Enhancement in a Non-fluorescent Nano-aggregated Fluorine-Boron-Based Dye

Marco Deiana,[1] Karam Chand,[2] Erik Chorell[2*] and Nasim Sabouri[1*]

[1] Department of Medical Biochemistry and Biophysics, Umeå University, 90187 Umeå, Sweden

[2] Department of Chemistry, Umeå University, 90187 Umeå, Sweden

AUTHOR INFORMATION

*Corresponding Authors:

erik.chorell@umu.se

nasim.sabouri@umu.se

Synthesis and characterization

Experimental

All reagents and solvents were used as such received from commercial suppliers unless stated otherwise. TLC was performed on aluminum backed silica gel plates (median pore size 60 Å, fluorescent indicator 254 nm) and detected with UV light. DMF was dried in a solvent drying system (activated molecular sieves in combination with an isocyanate scrubber). ^{1}H and ^{13}C NMR spectra were recorded on a Bruker 400 spectrometer at 298 K and calibrated by using the residual peak of the solvents as the internal standard (DMSO-d_6: δ H = 2.50 ppm; δ C = 39.50 ppm and $CDCl_3$: δ H = 7.26 ppm; δ C = 77.02 ppm). The abbreviations used in the NMR data are mentioned as singlet = s, doublet = d, triplet = t, multiplet = m, doubledoublet = dd, and broad singlet = brs. LC-MS was conducted on an Agilent 6150 Series Quadrupole LC/MS system. HRMS was performed by using a Agilent 1290 binary LC System connected to a Agilent 6230 Accurate-Mass TOF LC/MS (ESI+); calibrated with Agilent G1969-85001 ESTOF Reference Mix containing ammonium trifluoroacetate, purine and hexakis (1H, 1H, 3H tetrafluoropropoxy) phosphazine in 90:10 CH_3CN:H_2O.

Procedure for the preparation of benzimidazole–iminocoumarin compound (**2**)**:** To the mixture of 8-hydroxyjulolidine-9-carboxaldehyde **1** (750 mg, 3.5 mmol) and 2-benzimidazoleacetonitrile (623 mg, 3.9 mmol) in anhydrous ethanol (30 ml) was added catalytic amount (1-2 drops) of piperidine. After addition of piperidine, the reaction mixture became a clear solution within 30 min and the reaction was stirred for 12h. The re-appearance of precipitates in the reaction mixture indicate formation of product and progress of reaction was monitored on TLC until consumption of all starting materials. The precipitates were filtered and washed with ethanol under reduced pressure to give a brown-colored residue of benzimidazole-iminocoumarin derivative which was purified through crystallization in DMSO to give the pure desired compound **2** (812 mg) in 66% yield. ^{1}H NMR (400MHz, chloroform-*d*) δ (ppm): 8.03 (brs, 1H), 8.01 (d, *J* = 8.0 Hz, 1H), 7.89 (d, *J* = 8.0 Hz, 1H), 7.47 (t, *J* = 8.0 Hz, 1H), 7.35 (t, *J* = 8.0 Hz, 1H), 6.89 (s, 1H), 3.27-3.31 (m, 4H), 2.88 (t, *J* = 8.0 Hz, 2H), 2.75 (t, *J* = 8.0 Hz, 2H), 1.96-2.01 (m, 4H); ^{13}C NMR (100 MHz, chloroform-*d*) δ (ppm): 152.86, 150.72, 147.00, 137.81, 126.43, 126.03, 124.53, 122.32, 121.23, 117.90, 107.89, 105.95, 50.16, 49.75, 27.36, 21.40, 20.49, 20.22; m/z (HRMS): calculated for $C_{22}H_{20}N_3OS$ $(M+H)^+$: 374.1322; obtained 374.1340.

Procedure for the preparation of substituted benzimidazole-iminocoumarin compound (3a-b): To the mixture of benzimidazole-iminocoumarin **2** (400 mg, 1.12 mmol) and corresponding aniline (300 mg, 2.5 mmol) in 20 mL of ethanol, catalytic amount (18 mg, 10 mol%) of p-TsOH was added and the resulting mixture was refluxed overnight. Completion of reaction was monitored through LC-MS. On completion, solvent was cooled and then removed under reduced pressure. The crude so obtained was purified by column chromatography over silica (SiO_2), using 0-1.5% methanol in dichloromethane as eluent.

(Z)-10-(1*H*-benzo[d]imidazol-2-yl)-*N*-phenyl-2,3,6,7-tetrahydro-1*H*,5*H*,11*H*-pyrano[2,3-f]pyrido[3,2,1-ij]quinolin-11-imine (**3a**): The title compound (**3a**) was obtained from the reaction of **2** with aniline (by following the general procedure) as a red solid in 78% (378 mg) yield. ^{1}H NMR (400 MHz, chloroform-*d*) δ: 12.81 (s, 1H), 8.76 (s, 1H), 7.88 – 7.68 (m, 1H), 7.68 – 7.49 (m, 1H), 7.44 (dd, *J* = 8.3, 7.3 Hz, 2H), 7.36 – 7.32 (m, 2H), 7.28 (s, 2H), 7.19 (tt, *J* = 7.2, 1.3 Hz, 1H), 6.98 (s, 1H), 3.28 (t, *J* = 5.7 Hz, 2H), 3.24 (t, *J* = 5.7 Hz, 2H), 2.78 (t, *J* = 6.3 Hz, 2H), 2.57 (t, *J* = 6.4 Hz, 2H), 2.03 – 1.94 (m, 2H), 1.94 – 1.84 (m, 2H); ^{13}C NMR (100 MHz, DMSO-*d₆*) δ: 150.06, 149.47, 148.71, 146.53, 146.41, 137.59, 128.93, 126.59, 123.98, 123.72, 122.14, 118.37, 110.45, 107.96, 105.84, 49.81, 49.24, 27.27, 21.34, 20.35, 19.96; m/z (LC-MS): calculated for $C_{28}H_{24}N_4O$ $(M+H)^+$: 433.20; obtained 433.2.

(*Z*)-10-(1*H*-benzo[d]imidazol-2-yl)-*N*-(4-morpholinophenyl)-2,3,6,7-tetrahydro-1*H*,5*H*,11*H*-pyrano[2,3-f]pyrido[3,2,1-ij]quinolin-11-imine (3b): The title compound (**3b**) was obtained from the reaction of **2** (400 mg) with 4-morpholinoaniline (440 mg) by following the general procedure as a red solid in 69% (401 mg) yield. ^{1}H NMR (400 MHz, DMSO-d_6) δ: 12.51 (s, 1H), 8.47 (s, 1H), 7.70 (s, 1H), 7.61 (d, *J* = 4 Hz, 1H), 7.44-7.43 (m, 2H), 7.18-7.17 (m, 2H), 7.12 (s, 1H), 7.00 – 6.99 (m, 2H), 3.79 – 3.78 (m, 4H), 3.30 – 3.26 (m, 4H), 3.16 – 3.14 (m, 4H), 2.73 (t, *J* = 4.0 Hz, 2H), 2.67 (t, *J* = 4.0 Hz, 2H), 1.92-1.87 (m, 4H); ^{13}C NMR (100 MHz, chloroform-d) δ: 157.71, 150.24, 149.16, 148.09, 146.64, 138.53, 137.57, 137.20, 126.38, 124.40, 122.49, 120.56, 118.56, 116.12, 108.50, 106.12, 66.98, 50.17, 49.90, 49.64, 27.37, 21.39, 20.60, 20.33; m/z (LC-MS): calculated for $C_{32}H_{31}N_5O_2$ $(M+H)^+$: 518.2; obtained 518.2.

General procedure for preparation of benzimidazole–iminocoumarin based bodipy complexes (4a-b):

To the mixture of benzimidazole–iminocoumarin (300 mg, 0.69 mmol) in dichloroethane, was added diisopropylamine (0.3 mL, 1.7 mmol) and $BF_3.OEt_2$ (0.23 mL, 1.7 mmol) and the resulting mixture was stirred at 90 °C for 1-2 hours. Completion of the reaction was monitored through LC-MS. On completion, the reaction mixture was allowed to attain room temperature. The cooled mixture was diluted with dichloromethane and washed with a saturated solution of $NaHCO_3$. The organic layers were combined, dried over $MgSO_4$ and the solvent was removed under reduced pressure. The crude mixture so obtained was purified through column chromatography in silica (SiO_2) with 0-2% methanol in dichloromethane as eluent.

6,6-Difluoro-7-phenyl-8b,8b1,10,11,14,15-hexahydro-6*H*,9*H*,13*H*-6λ4,7λ4-benzo[4'',5'']imidazo[1'',2'':3',4'][1,3,2]diazaborinino[5',6':5,6]pyrano[2,3-f]pyrido[3,2,1-ij]quinoline (4a): The title compound (**4a**) was obtained from the reaction of **3a** with boron trifluoride etherate by following the general procedure as a red solid in 73% (243mg) yield. ^{1}H NMR (400 MHz, Chloroform-*d*) δ: 8.97 (s, 1H), 7.73 (dd, *J* = 8.0 & 4.0 Hz, 2H), 7.57 – 7.53 (m, 4H), 7.47 (t, *J* = 4.0 Hz, 1H), 7.25 – 7.20 (m, 4H), 7.10 (s, 1H), 3.30 (t, *J* = 4.0 Hz, 2H), 3.24 (t, *J* = 4.0 Hz, 2H), 2.77 (t, *J* = 4.0 Hz, 2H), 2.18 (t, *J* = 4.0 Hz, 2H), 1.98-1.92 (m, 2H), 1.81-1.77 (m, 2H). ^{13}C NMR (100 MHz, Chloroform-*d*) δ: 159.34, 148.63, 145.88, 140.02, 136.96, 136.09, 129.28, 128.14, 127.08, 122.69, 122.52, 121.95, 117.45, 113.93, 109.21, 105.85, 50.32, 49.77, 27.38, 20.72, 19.70, 18.98; m/z (HRMS): cald. for $C_{28}H_{24}BF_2N_4O^+$ $(M + H)^+$: 481.2006; obtained 481.2037.

6,6-difluoro-7-(4-morpholinophenyl)-8b,8b1,10,11,14,15,17,18-octahydro-6H,9H,13H-6l4,7l4-benzo[4'',5'']imidazo[1'',2'':3',4'][1,3,2]diazaborinino[5',6':5,6]pyrano[2,3-f]pyrido[3,2,1-ij]quinoline (4b): The title compound (**4b**) was obtained from the reaction of **3b** with boron trifluoride etherate by following the general procedure as a red solid in 58% (190 mg) yield. ^{1}H NMR (400 MHz, Chloroform-*d*) δ: 9.11 (s, 1H), 7.83 – 7.76 (m, 2H), 7.41 – 7.32 (m, 4H), 7.29 (s, 1H), 7.10 – 7.01 (m, 2H), 3.95 – 3.88 (m, 4H), 3.37 (t, J = 5.8 Hz, 2H), 3.32 (t, J = 5.8 Hz, 2H), 3.28 – 3.21 (m, 4H), 2.79 (t, *J* = 6.3 Hz, 2H), 2.30 (t, *J* = 6.4 Hz, 2H), 2.01 – 1.93 (m, 2H), 1.88 – 1.81 (m, 2H); ^{13}C NMR (100 MHz, Chloroform-*d*) δ 158.41, 151.17, 150.17, 149.96, 144.30, 141.51, 134.19, 128.36, 128.00, 127.47, 124.80, 124.29, 122.67, 115.97, 115.42, 114.74, 109.59, 106.02, 66.80, 50.68, 50.07, 49.20, 27.25, 20.59, 19.64, 19.07; m/z (HRMS): cald. for $C_{32}H_{31}BF_2N_5O_2^+$ $(M + H)^+$: 566.2533; obtained 566.2555.

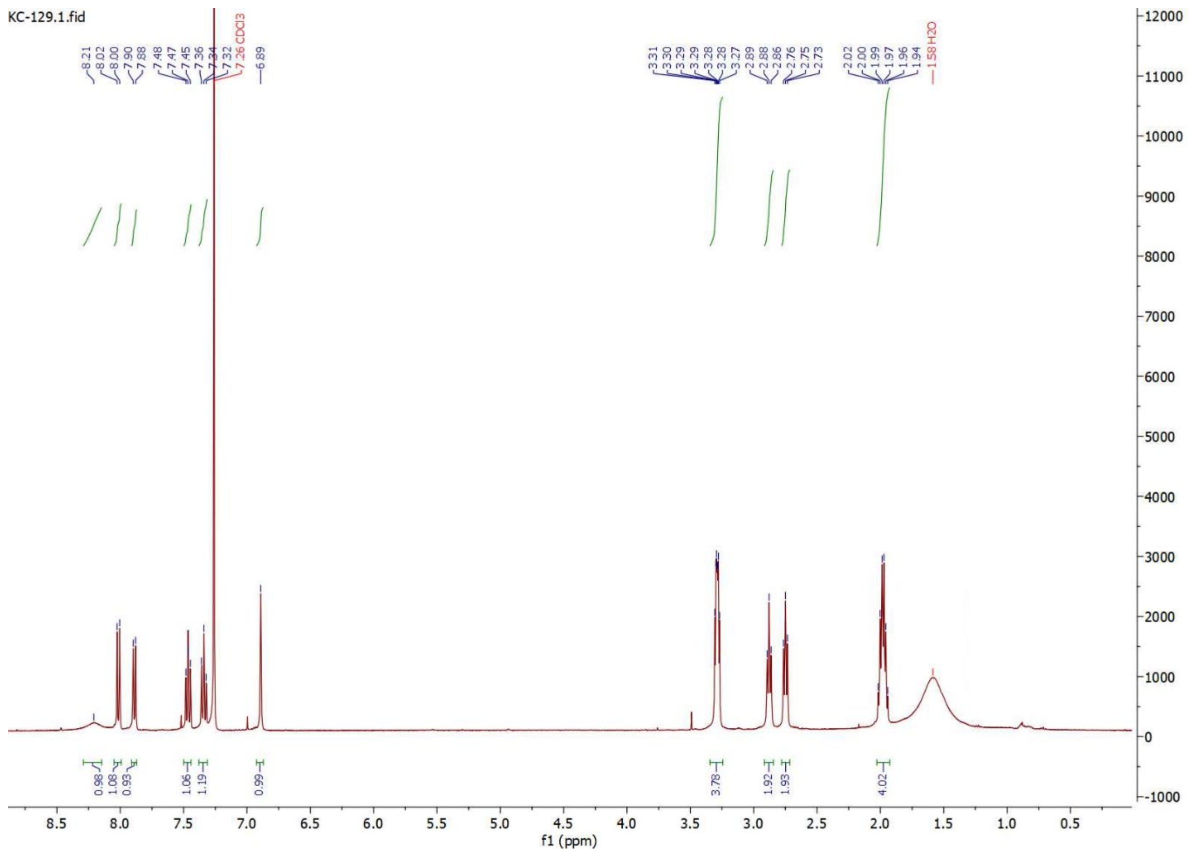

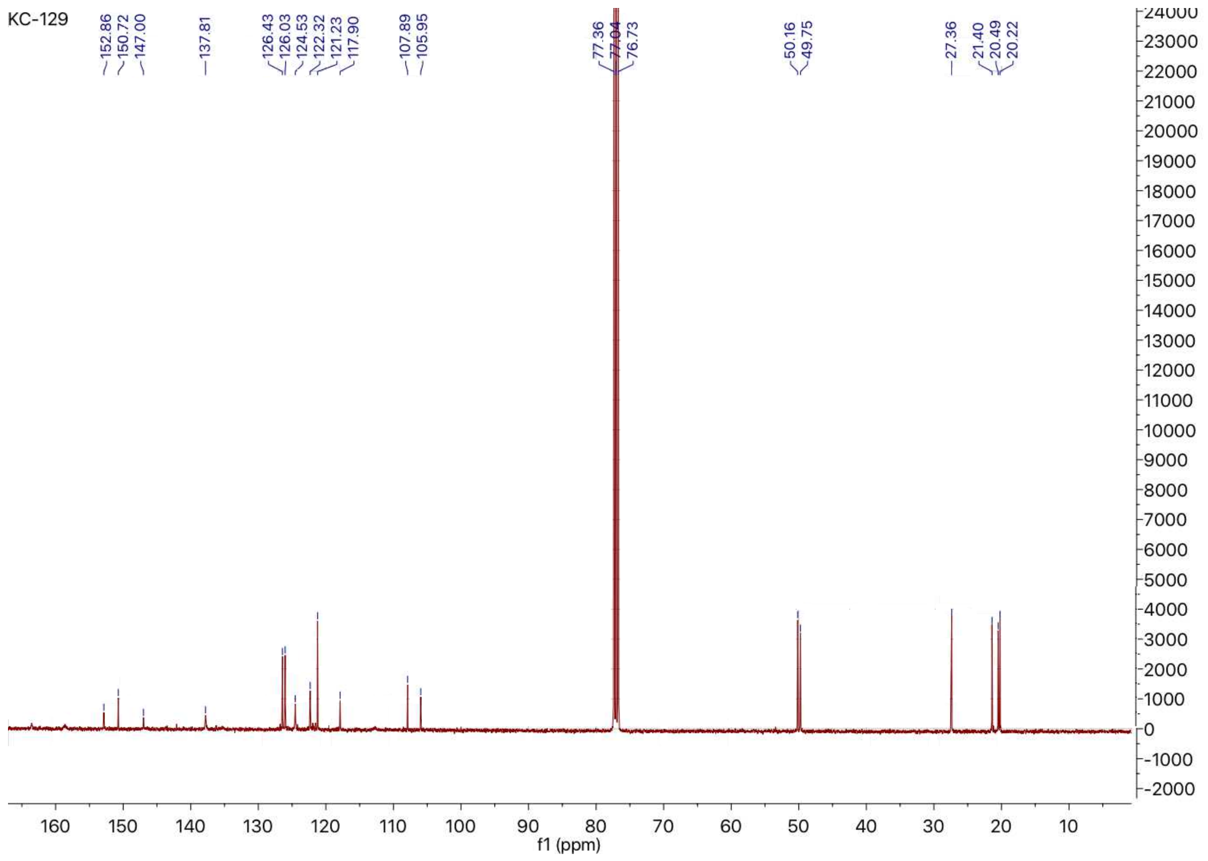

Figure S1. ^{1}H & ^{13}C NMR spectra of compound **2**.

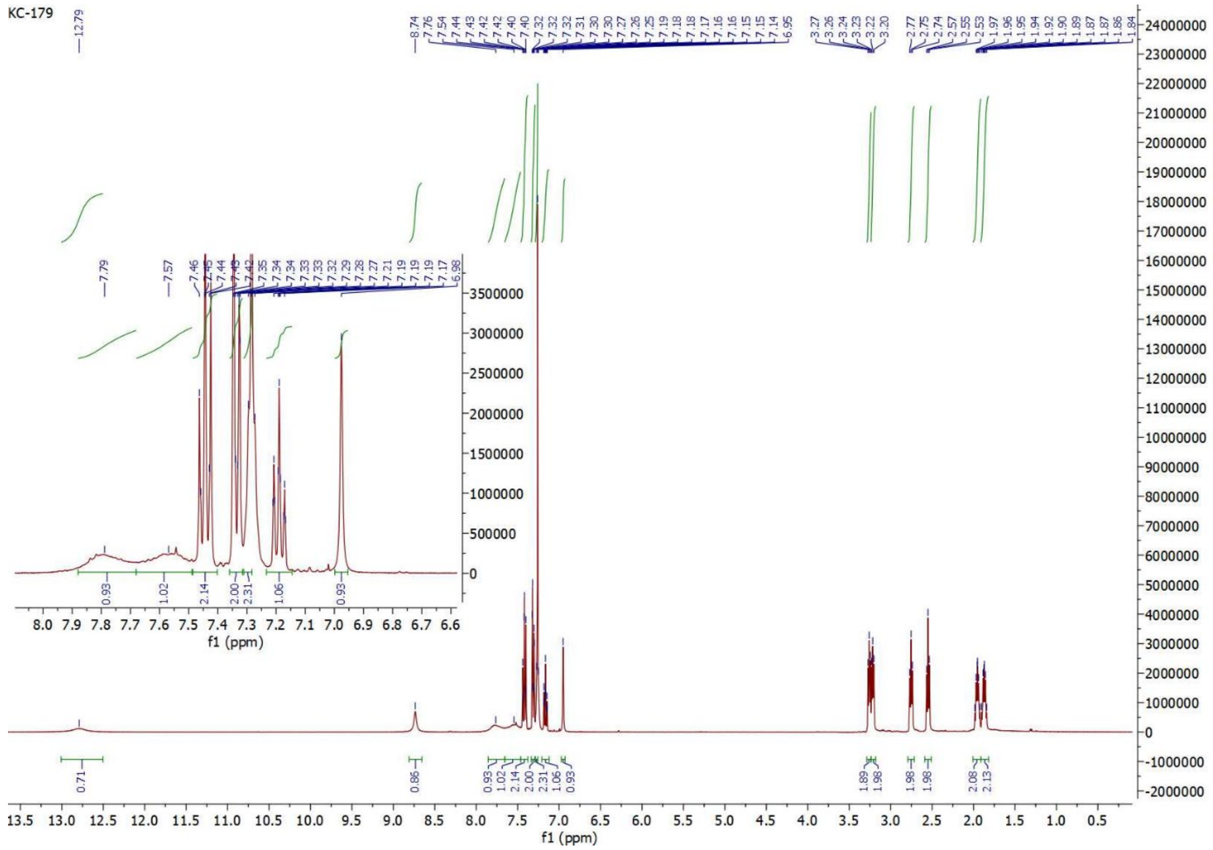

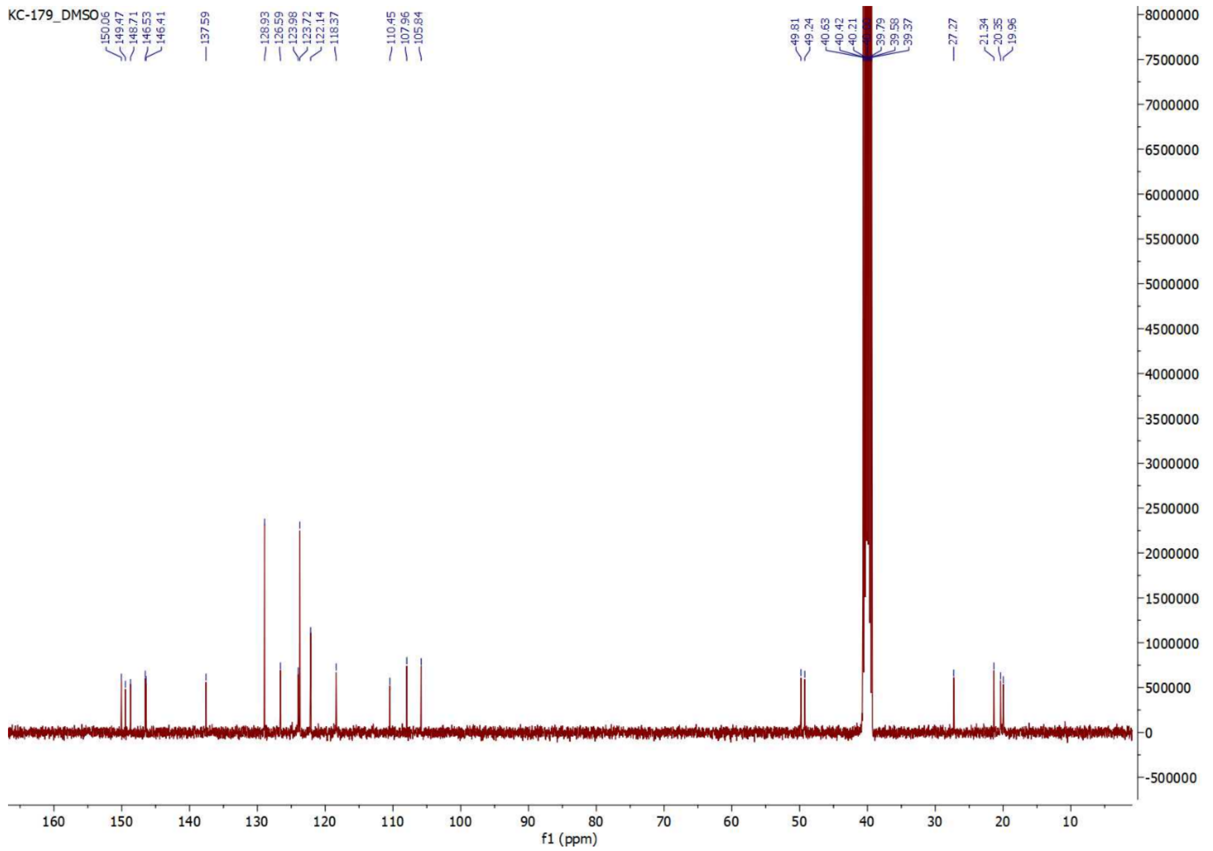

Figure S2. ^{1}H & ^{13}C NMR spectra of compound **3a**.

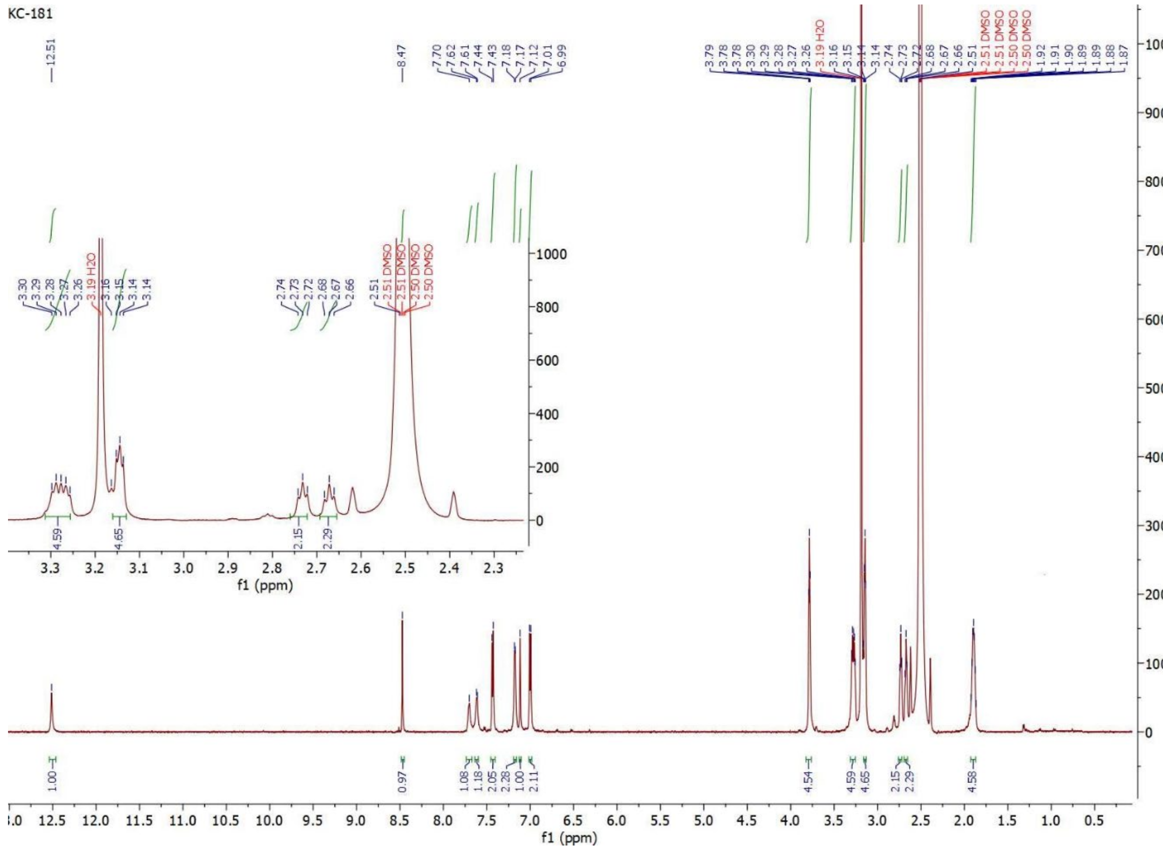

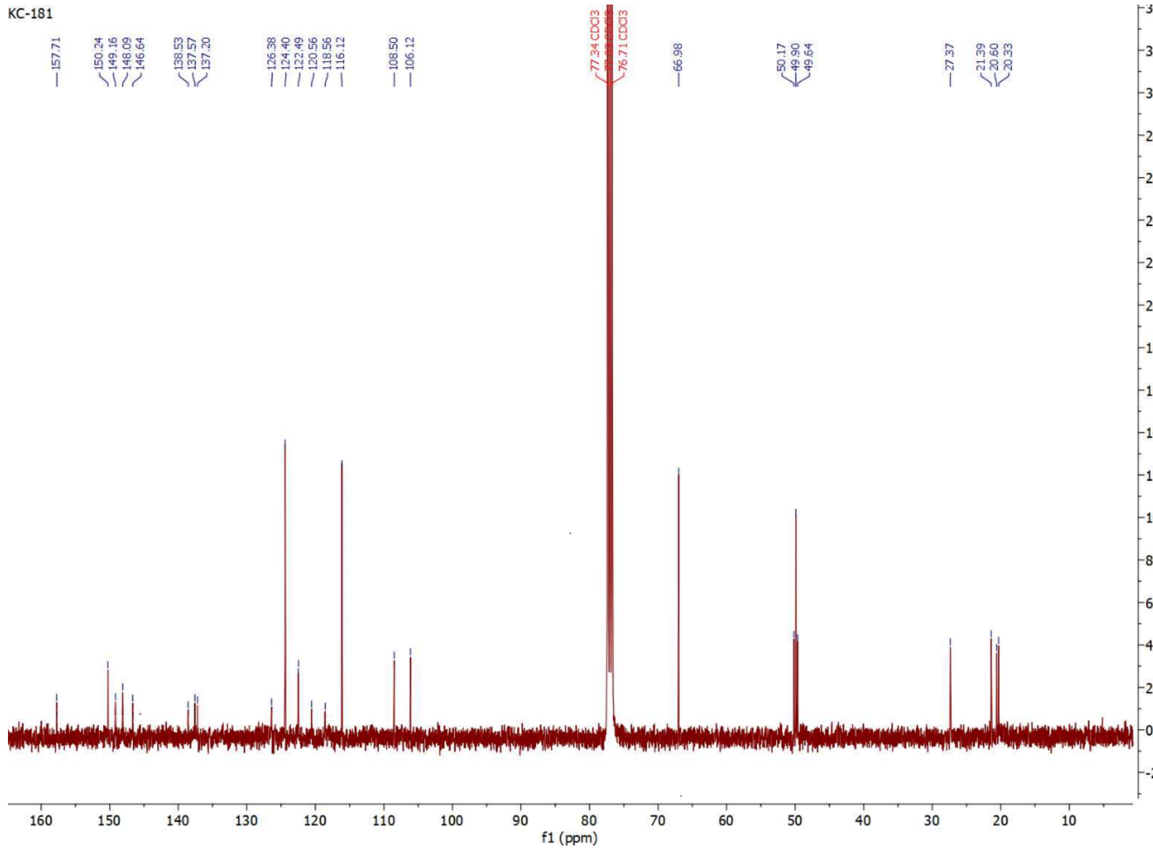

Figure S3. ^{1}H & ^{13}C NMR spectra of compound **3b**.

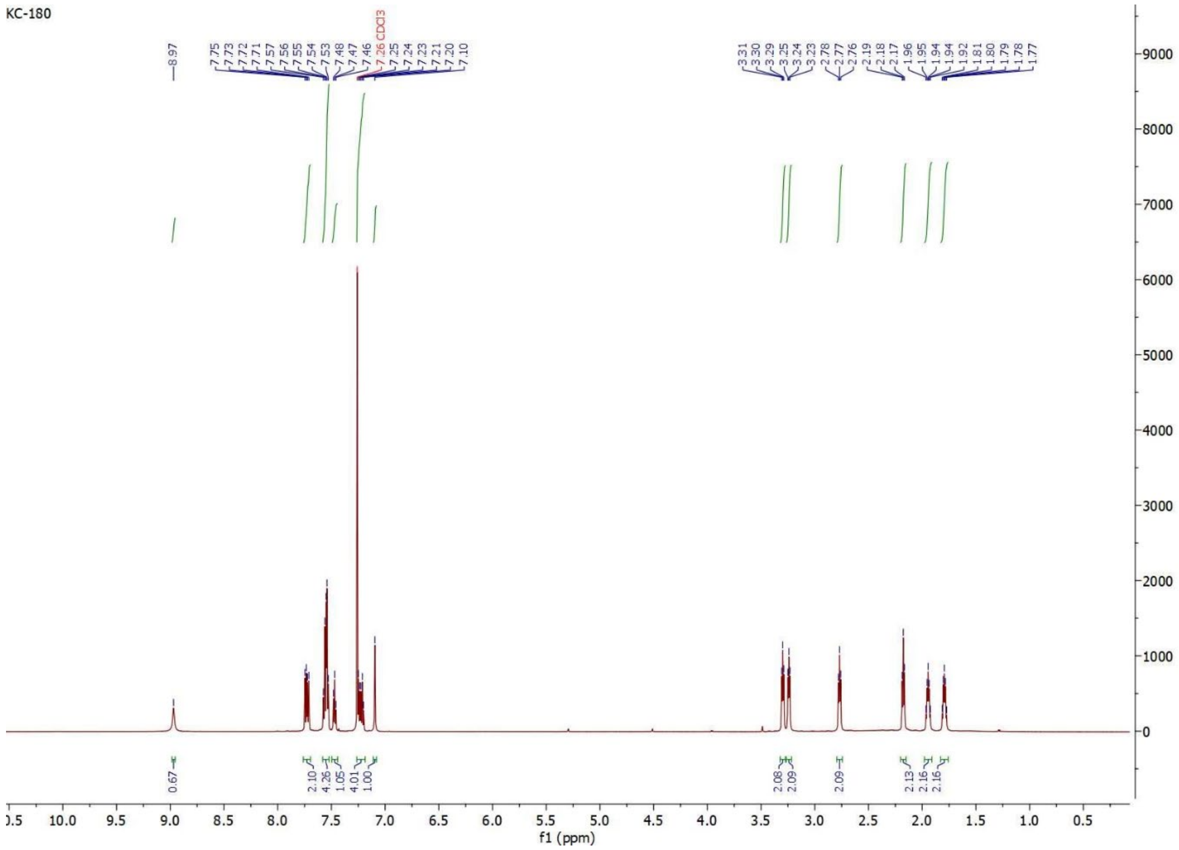

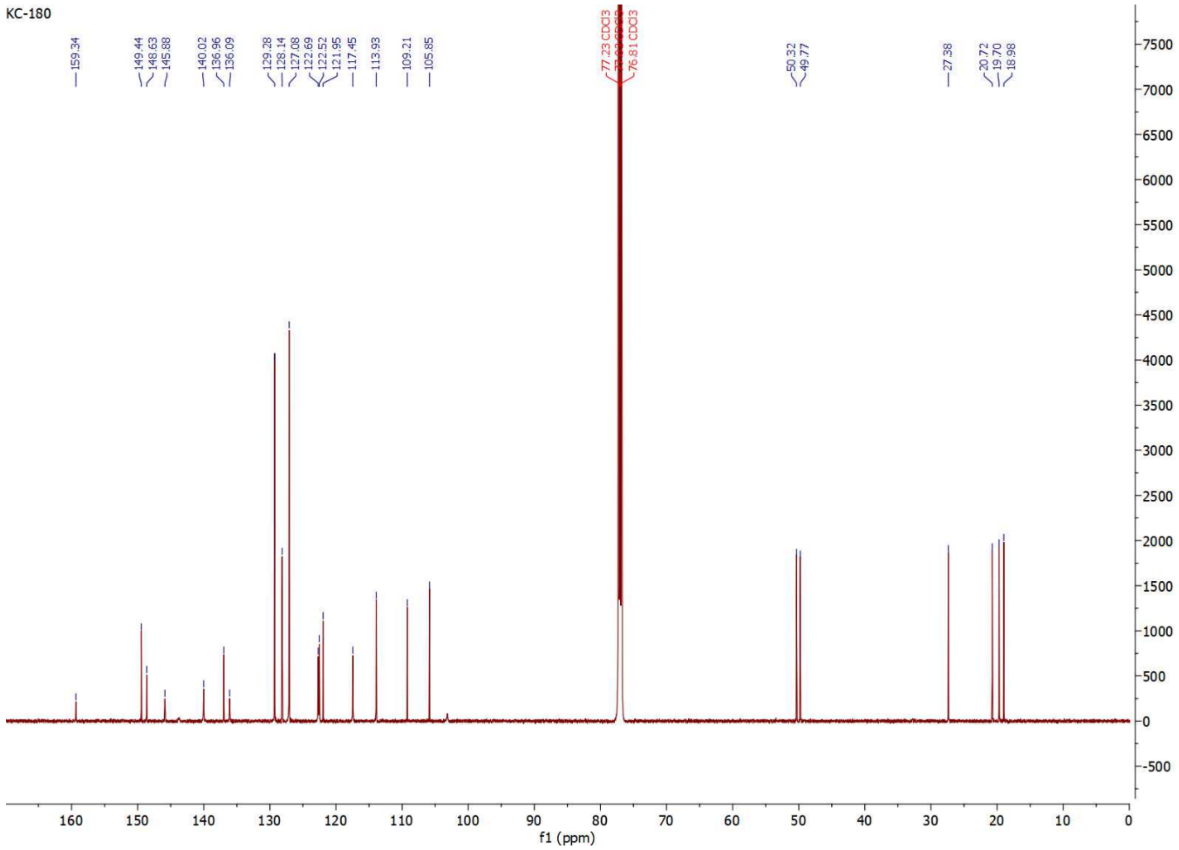

Figure S4. ^{1}H & ^{13}C NMR spectra of compound **4a**.

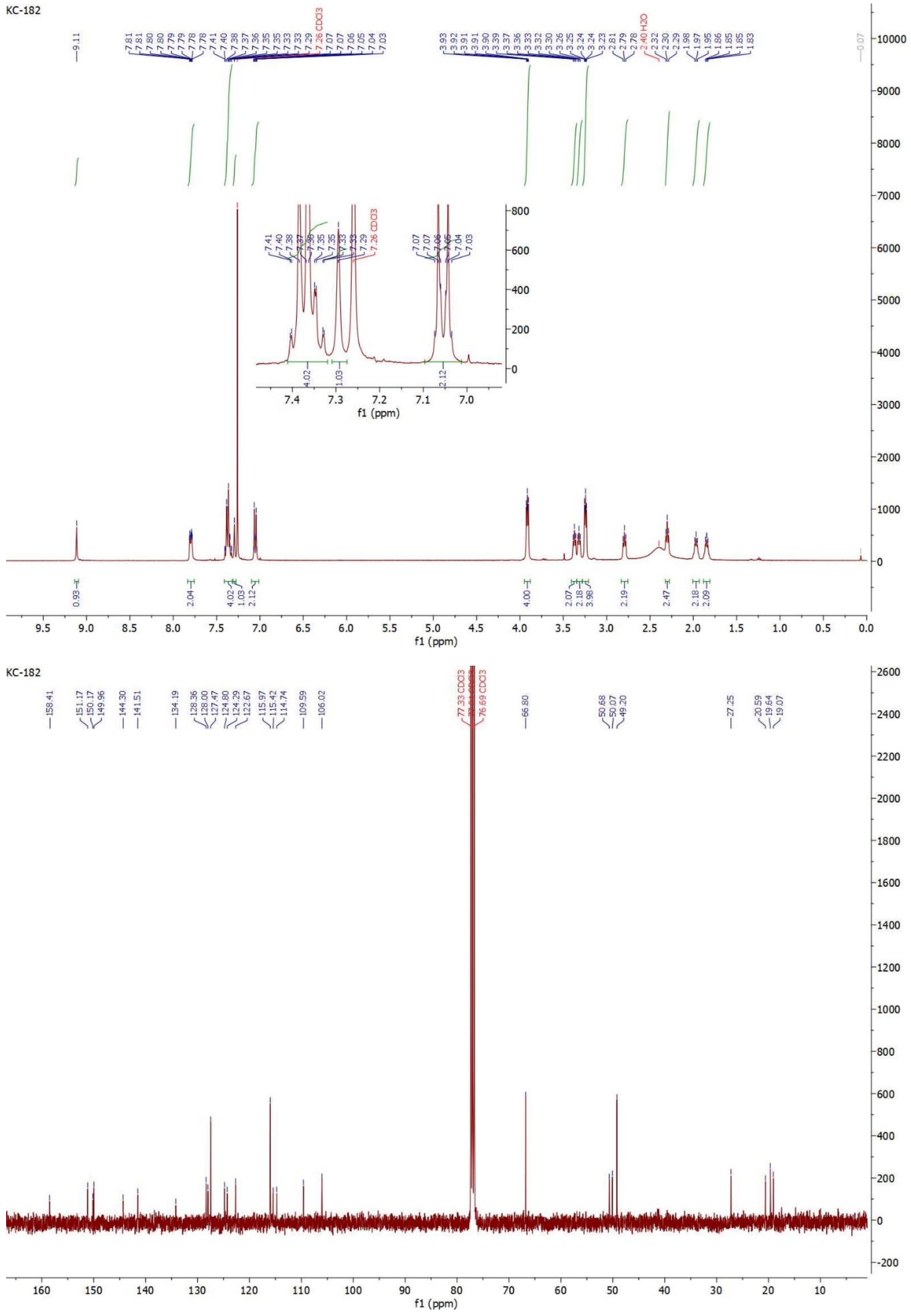

Figure S5. ^{1}H & ^{13}C NMR spectra of compound **4b**.

Experimental procedures

General

Solvents, reagents, chemicals and oligonucleotides were purchased from commercial suppliers (Sigma-Aldrich and Eurofins Genomics) and used without further modifications, unless otherwise stated. Oligonucleotides were diluted with ultrapure water and stored at - 20 °C. The exact oligonucleotide concentration was determined by UV/Vis spectroscopy using the molar extinction coefficients (ε_{260}) provided in Table S1 or S2 and calculated by using oligo analyzer on the IDT web site. A 1 M KCl stock solution was prepared by using solid KCl. The aqueous stock solution (1 M) of Tris buffer was prepared in water and the pH was then adjusted to 7.4. The stock solutions of the synthesized compounds were prepared in DMSO. The final concentration of DMSO in all the DNA-based assays reached the maximum value of 4.0 % (v/v).

Optical spectroscopy

CD measurements were recorded on a Jasco J-1700 CD spectrometer at 25 °C using a cell length of 1 cm, and over a wavelength range of 220-320 nm. CD spectra were baseline-corrected from the buffer. UV/Vis absorption spectra were recorded on T90+ UV/Vis spectrometer (PG instruments Ltd). The spectral band width was 1 nm. Steady-state emission spectra and kinetic curves were recorded on Jasco FP-6500 spectrofluorometer equipped with the JascoPeltier-type temperature controller (ETC2736).

Atomic force microscopy (AFM)

AFM imaging was carried out by using a BioScope Catalyst atomic force microscope (Bruker) in peak force tapping mode in air. Resolution was set at 512 × 512 pixels, scan size was 1 μm, and scan rate was 1.0 Hz. Peak force set point and gain were controlled automatically. Bruker ScanAsyst-Air cantilevers were used in all measurements. Samples were deposited on the

surface of freshly cleaved mica (Ted Pella) for 15 min, washed three times with 100 μL deionized water, and dried at room temperature.

Dynamic Light Scattering (DLS)

DLS was performed by using a MAVERN particle size analyzer Nano S instrument. Samples were prepared at 1 μM concentration by diluting them from the DMSO stock solutions into water (DMSO 0.1 % and 0.2 % for **4b** and **4a**, respectively). Before analysis the samples were left to equilibrate for 2 hours. Particle size is provided as the average of three independent measurements.

Photometric and fluorimetric titrations

The freshly prepared **4b** solution (c_{4b} = 3 μM, c_{TRIS} = 50 mM, pH: 7.4, c_{KCl} = 100 mM) was titrated with the oligonucleotides and let to equilibrate for several minutes before recording the UV/Vis or emission spectra. The concentration of the experiments was optimized to have an optical density < 0.15 to avoid reabsorption in the fluorescence emission. The excitation wavelength was set at the isosbestic point centered at 513 nm.

Light-up response of 4b complexed with mitochondrial DNA G4 sequences

The freshly prepared **4b** solution (c_{4b} = 1 μM, c_{TRIS} = 50 mM, pH: 7.4, c_{KCl} = 100 mM) was titrated with the mtDNA G4s (see Table S3) (c_{G4s} = 2 μM) and let to equilibrate for 30 minutes before recording the emission spectra. The excitation wavelength was set at the isosbestic point centered at 513 nm. Fold change in fluorescence intensity is defined as the ratio between **4b**-mt DNA G4 system (F) over **4b** emission intensity (F_0).

Fluorescence displacement assay

Fluorescence displacement assay was performed by titrating the **4b**-*c-KIT* 2 binary mixture with increasing concentration of the well-known G4-binders Phen-DC_3 or BRACO-19 until no change on the **4b**´s fluorescence intensity was observed.

Data processing

Binding constants were obtained with Bindfit[1, 2] by using multiple global fitting methods (Nelder-Mead method) of both the UV/Vis data in the range of 455 – 565 nm and fluorescence data in the range 530 – 610 nm. Dilution corrections was included in the fitting option. Fitted parameters with the 1:1 and 1:2 binding models are reported in Table S2.

Solvent-dependent studies

Solvent-dependent studies were performed by dissolving **4a** or **4b** in different organic solvents having different polarities. Then, the UV/Vis and the emission spectra were recorded by exciting the samples at their absorption maxima.

Aggregation studies

Disaggregation studies were carried out by titrating sodium dodecyl sulfate (SDS) into **4a** or **4b** aqueous solution at different concentrations. The optical density or the fluorescence intensity of the compounds were plotted as a function of the SDS concentration.

Nuclear Magnetic Resonance (NMR) studies

c-MYC Pu22 solution ($c_{c\text{-}MYC\ \mathrm{Pu22}}$ = 100 µM, c_{TRIS} = 10 mM, pH: 7.4, c_{KCl} = 100 mM, D_2O = 10 %) was titrated with different concentrations of **4b** (100, 200 and 400 µM taken from the 10 mM DMSO-d_6 stock solution). Control samples were also prepared by adding the same equivalent amount of DMSO-d_6 to the *c-MYC* Pu22 solution to verify that DMSO-d_6 did not have a significant effect on the G4 structure. All spectra were recorded in 3 mm NMR tubes at

298 K on a Bruker 850 MHz Avance III HD spectrometer equipped with a 5 mm TCI cryoprobe. Excitation sculpting was used in the ^{1}H NMR experiments, and 256 scans were recorded.

Cytotoxicity assay

HeLa cells were culture at 37 °C in 5% CO_2 in DMEM medium supplemented with penicillin-streptomycin (1×), and 10% fetal bovine serum. 5 × 10^3 cells/well were seeded in complete medium on 96 well-plates 24 h before the treatment with **4b**. **4b** was dissolved in complete medium at the indicated concentrations (DMSO reached the max value of = 1.0 % v/v) and added to cells. The status of the cells was monitored by using an EVOS® FL cell imaging system. At 48 h after **4b** treatment, PrestoBlue™ (Invitrogen, Ref No: A13261) was added to each well and the cells were incubated at 37 °C in 5% CO_2 for three additional hours. Cell viability was measured by recording the fluorescence signal of PrestoBlue ($\lambda_{exc}/\lambda_{em}$: 560/590 nm) using a Synergy H4 microplate reader (Biotek). N = 4 (mean ± SD).

Fluorescence microscopy

10 × 10^4 HeLa cells were seeded for 48h before treatment on a glass-bottomed microwell dish (MatTek Corp.). HeLa cells were washed with 1× PBS (2 times) and treated with **4b** (5 μM), the nuclear dye Hoechst 33342 (500 nM; Sigma-Aldrich, Ref No: B2261) and MitoTracker® Red CMXRos (100 nM, Invitrogen) dissolved in the live cell imaging solution (Molecular Probes™, Ref No: A14291DJ) for 15 min at 37 °C in 5% CO_2 prior imaging.

For fixed cell imaging, cells were fixed in cold methanol for 10 min and permeabilized with PBST (phosphate-buffered saline supplemented with 0.1% Triton X-100). Fixed cells were treated with **4b** (5 μM) for 30 min at RT. For the fluorescence competition assay, **4b** (5 μM) was incubated with BRACO-19 (5, 10 or 20 μM) for 30 min at RT.

Images were acquired using a Leica SP8 FALCON confocal microscope equipped with an incubation chamber operating at 37 °C in 5% CO_2. Maximum intensity projection of Z-stack images was used for data presentation. All data were processed by using ImageJ software.

Solvent-dependent absorption and emission studies

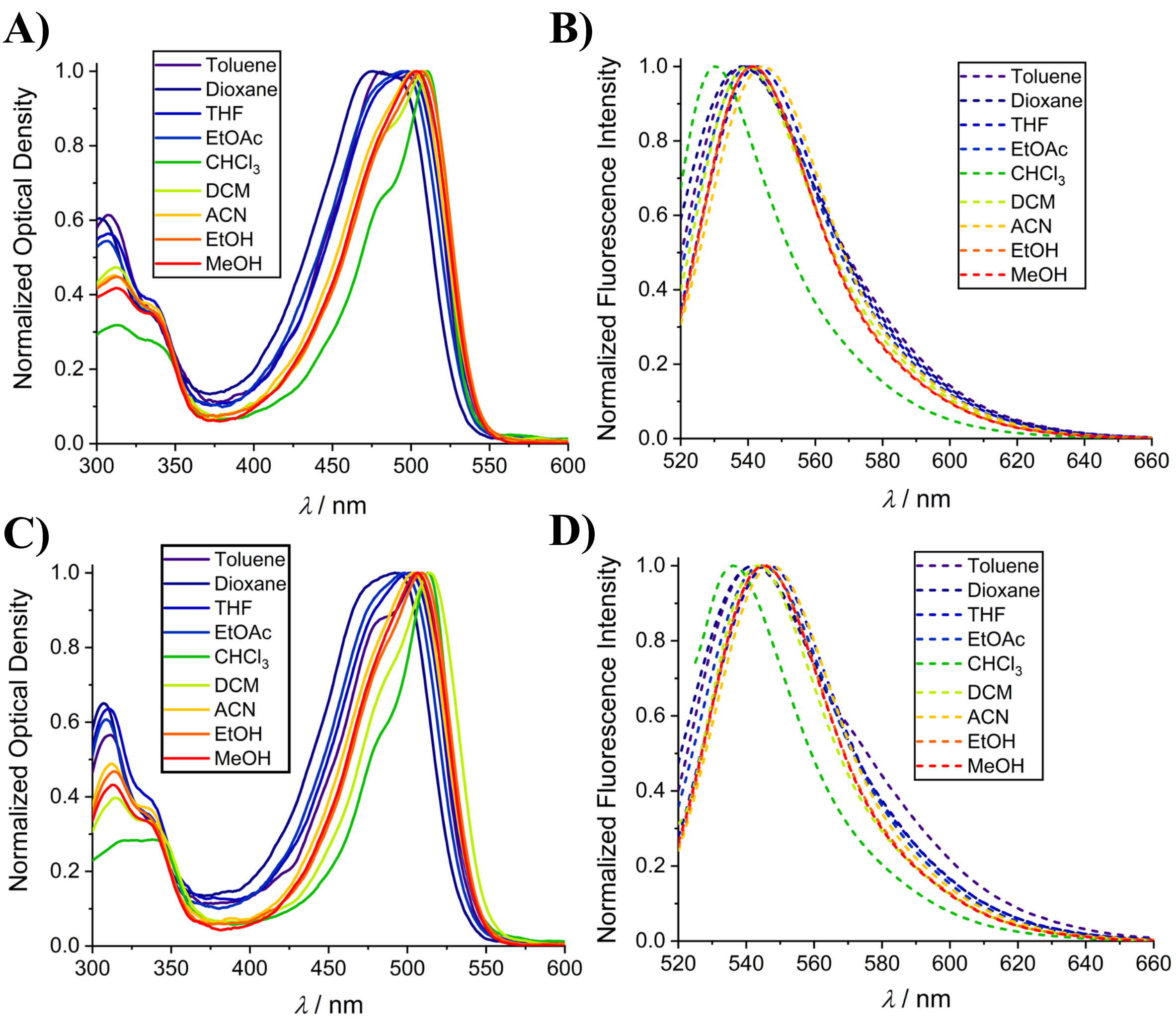

Figure S6. Solvent-dependent UV/Vis and emission spectra recorded for **4a** (A and B) and **4b** (C and D) in various organic solvents ($c_{\mathbf{4a}}$ and $c_{\mathbf{4b}}$ = 2 μM).

4a self-assembly properties

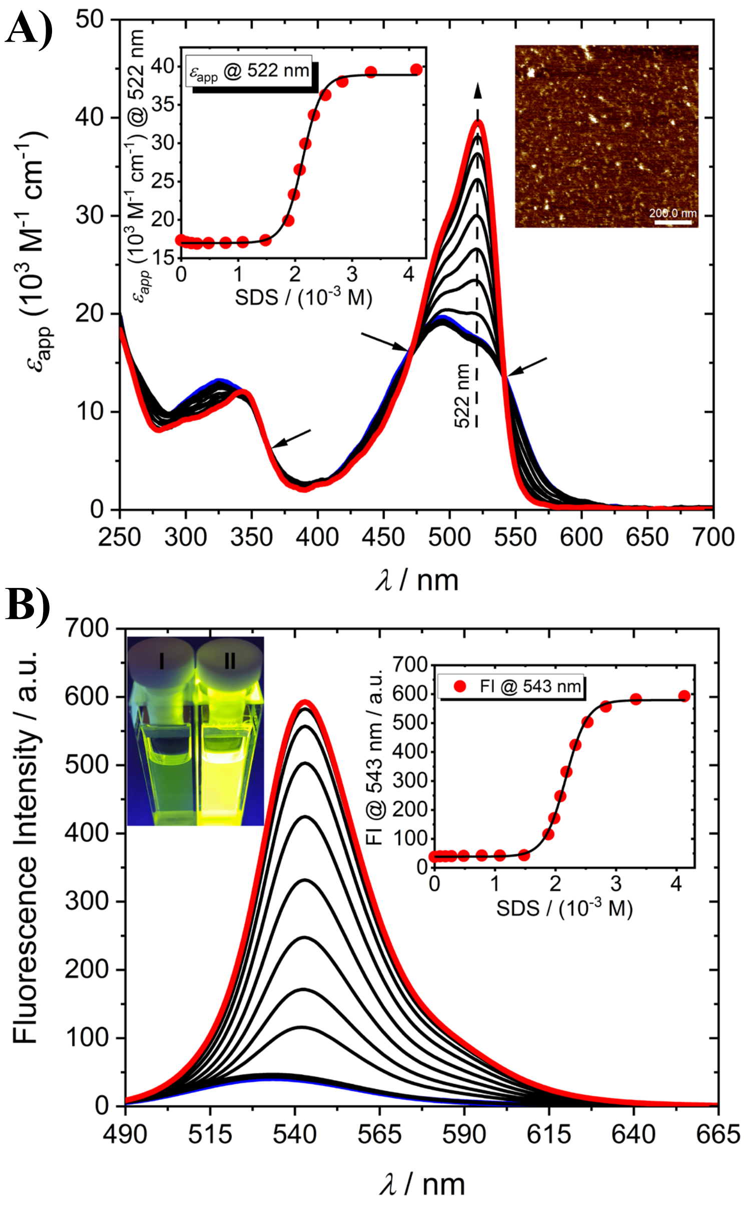

Figure S7. UV/Vis absorption (A) and steady-state emission (B) spectra of **4a** ($c_{\mathbf{4a}}$ = 3 µM, c_{TRIS} = 50 mM, pH: 7.4, blue line, λ_{exc} = 470 nm) with increasing concentrations of SDS (c_{SDS} = 0 to 4.13 mM). The insets in (A) show the spectral evolution profile of **4a** in the presence of SDS and a representative AFM height image of **4a**-nanoaggregates ($c_{\mathbf{4a}}$ = 10 µM, c_{TRIS} = 10 mM, pH: 7.4). The insets in (B) show the spectral evolution profile of **4a** in the presence of SDS and the resulting SDS-induced color change on **4a**.

Dynamic light scattering (DLS)

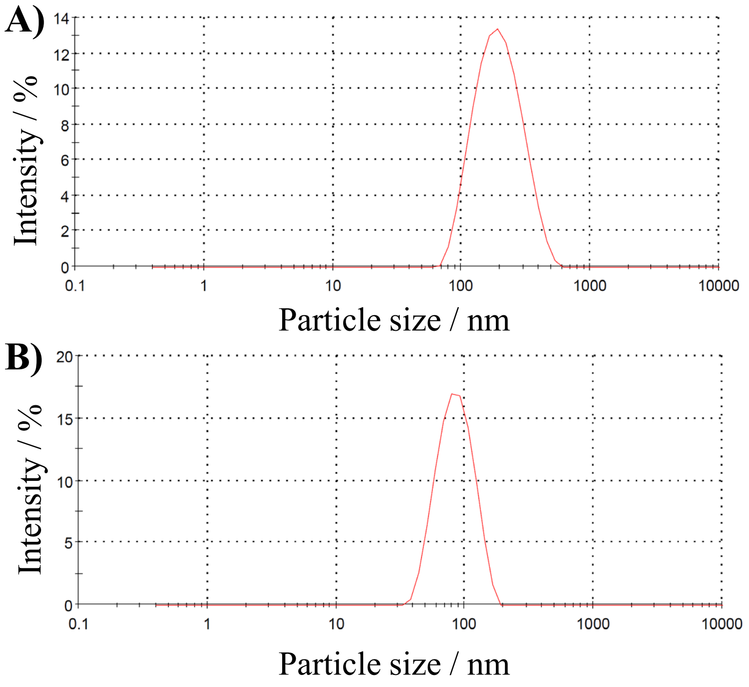

Figure S8. DLS studies of (A) **4a** and (B) **4b** in water (c_{4a} and c_{4b} = 1 µM).

4a and 4b optical properties

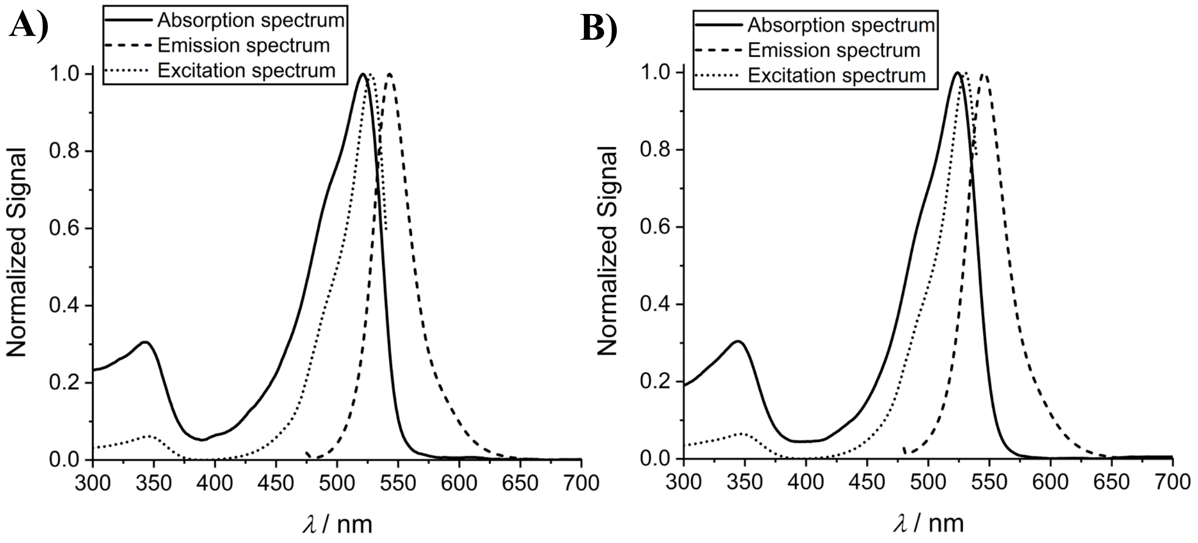

Figure S9. Normalized absorption (black solid line), emission (dashed line) and excitation (dotted line) spectra of **4a** (A) and **4b** (B) in the presence of SDS (c_{SDS} ~ 4 mM, $c_{4a\text{-}4b}$ = 3 µM, Tris buffer c = 50 mM, pH = 7.5). The absorption spectrum refers to the compounds´ monomeric band formed in the presence of SDS.

UV/Vis titration studies of 4b in the presence of parallel G4s

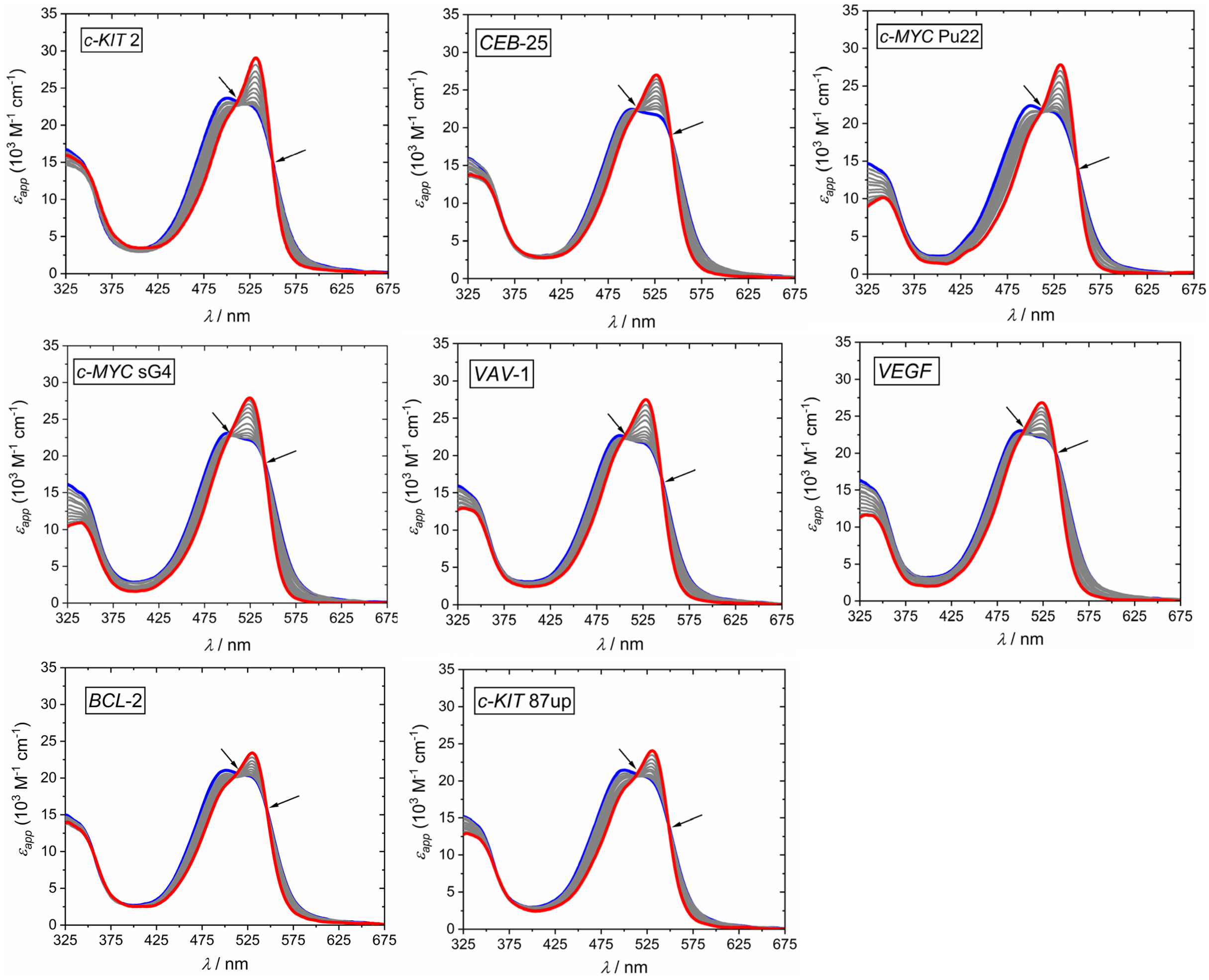

Figure S10. UV/Vis absorption spectra of **4b** in the absence (blue line) and presence (gray and red lines) of increasing concentrations of parallel G4s ($c_{\mathbf{4b}}$ = 3 µM, $c_{\text{parallel G4s}}$ = 0 to 17 µM, c_{KCl} = 100 mM, c_{TRIS} = 10 mM, pH: 7.4). The solid arrows indicate the appearance of isosbestic points. The sequences of the tested G4 oligonucleotides are shown in Table S1.

UV/Vis titration studies of 4a in the presence of parallel and hybrid G4s

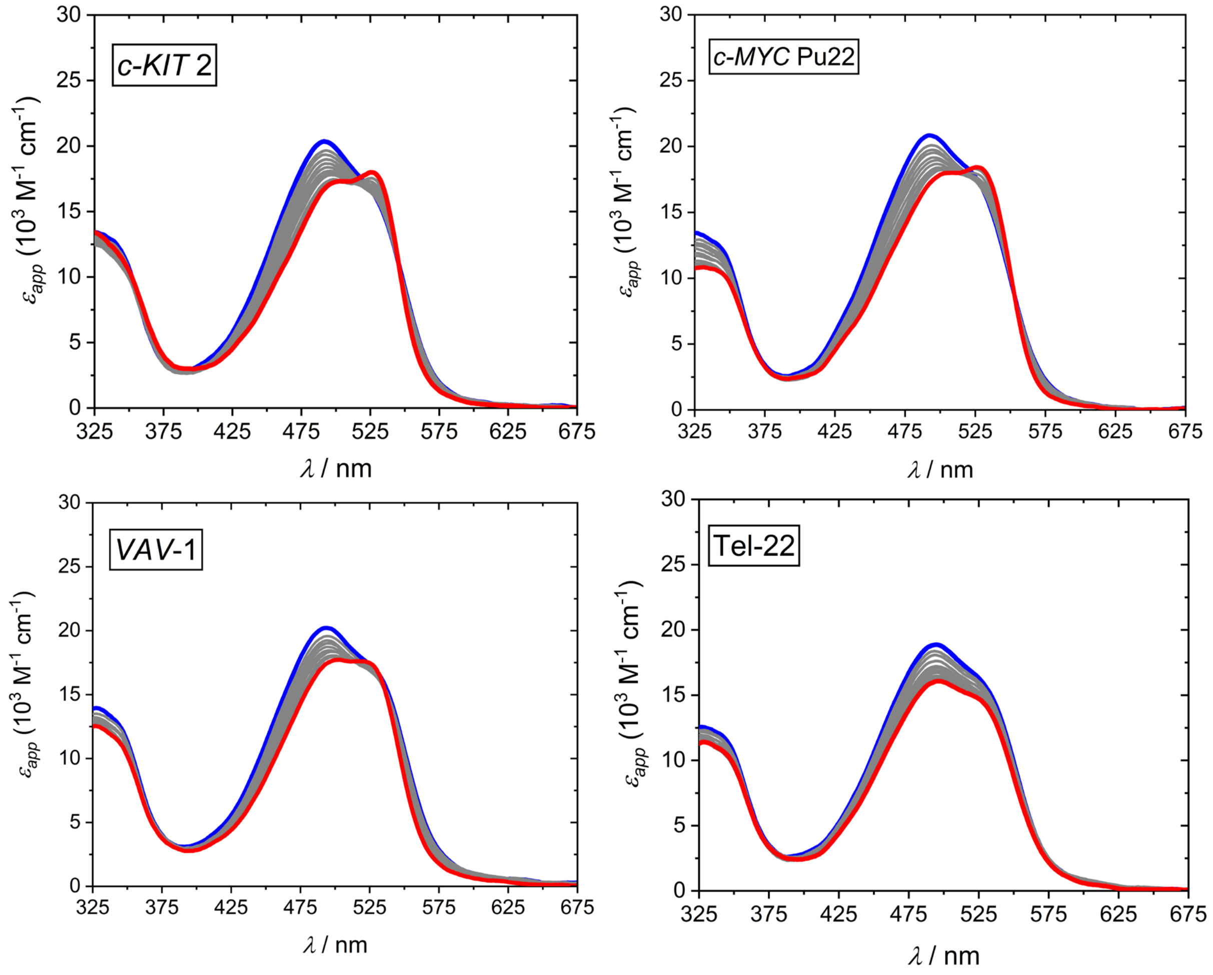

Figure S11. UV/Vis absorption spectra of **4a** in the absence (blue line) and presence (gray and red lines) of increasing concentrations of parallel and hybrid G4s ($c_{\mathbf{4a}}$ = 3 µM, $c_{\text{parallel/hybrid G4s}}$ = 0 to 17 µM, c_{KCl} = 100 mM, c_{TRIS} = 10 mM, pH: 7.4). The sequences of the tested G4 oligonucleotides are shown in Table S1.

Fluorescence titration studies of 4b in the presence of parallel G4s

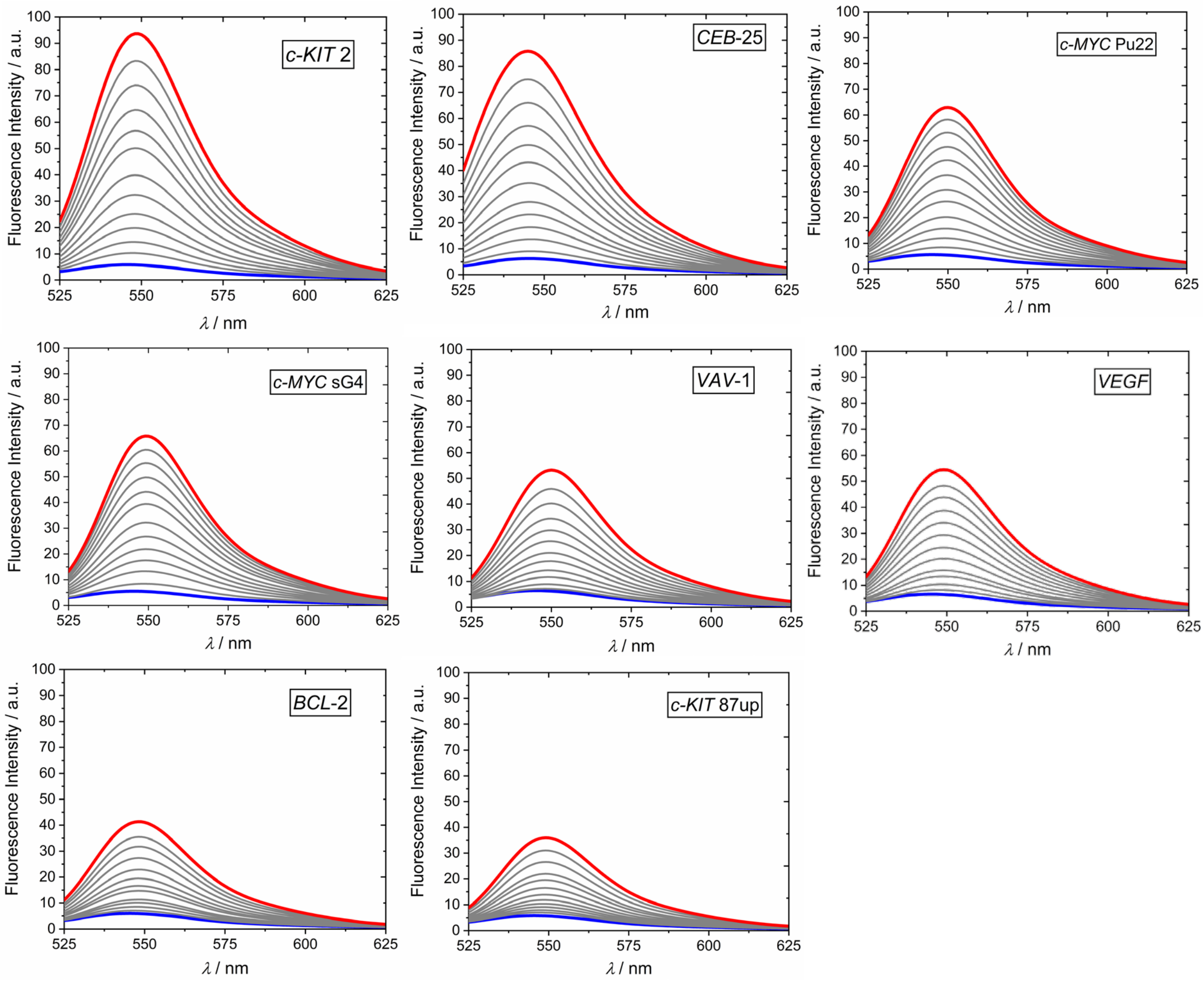

Figure S12. Steady-state emission spectra of **4b** in the absence (blue line) and presence (gray and red lines) of increasing concentrations of parallel G4s ($c_{\mathbf{4b}}$ = 3 µM, $c_{\text{parallel G4s}}$ = 0 to 21 µM, c_{KCl} = 100 mM, c_{TRIS} = 10 mM, pH: 7.4, λ_{exc} = 513 nm).

Fluorescence titration studies of 4b in the presence of hybrid, antiparallel and non-G4s

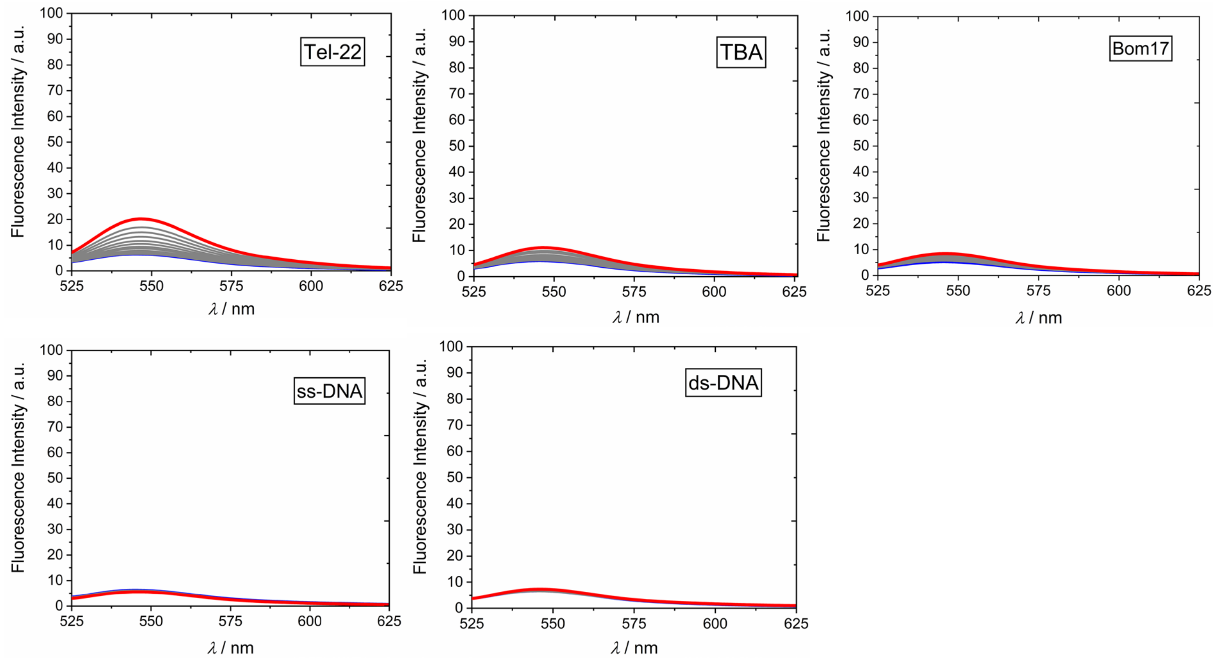

Figure S13. Steady-state emission spectra of **4b** in the absence (blue line) and presence (gray and red lines) of increasing concentrations of hybrid and antiparallel G4s ($c_{\mathbf{4b}}$ = 3 µM, $c_{\text{hybrid/antiparallel G4s}}$ = 0 to 21 µM, c_{KCl} = 100 mM, c_{TRIS} = 10 mM, pH: 7.4, λ_{exc} = 513 nm).

UV/Vis titration studies of 4b in the presence of hybrid, antiparallel and non-G4s

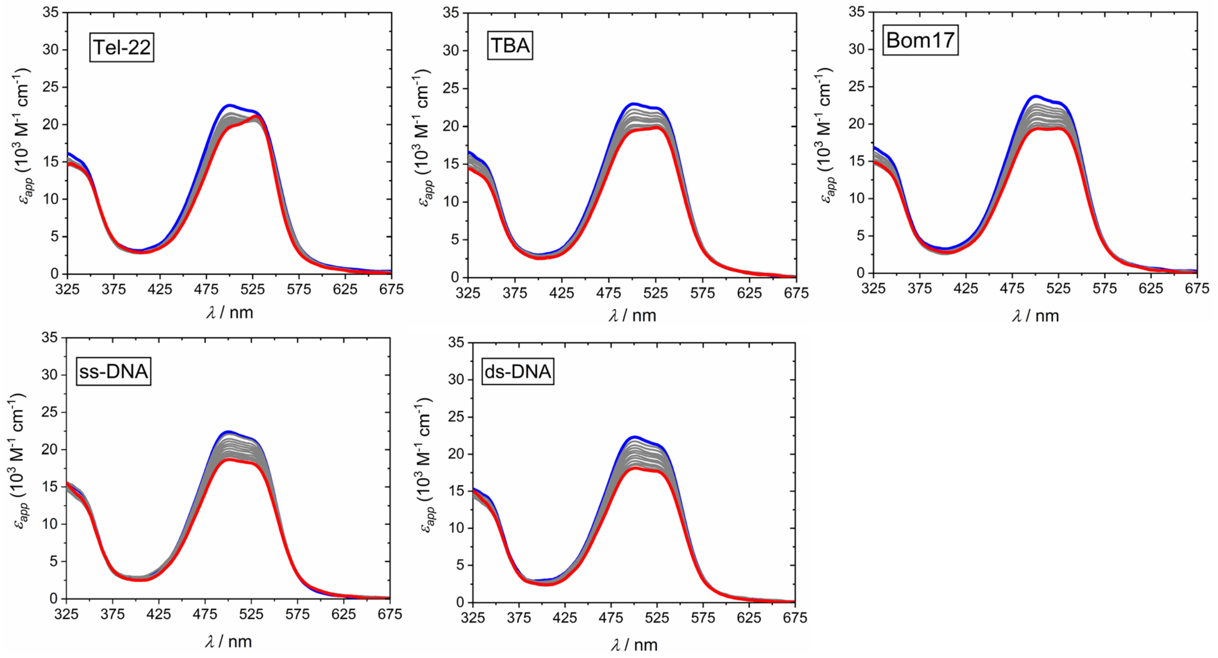

Figure S14. UV/Vis absorption spectra of **4b** in the absence (blue line) and presence (gray and red lines) of increasing concentrations of hybrid, antiparallel and non-G4s ($c_{\mathbf{4b}}$ = 3 µM, $c_{\text{hybrid/antiparallel/non-G4s}}$ = 0 to 17 µM, c_{KCl} = 100 mM, c_{TRIS} = 10 mM, pH: 7.4).

Effects of pH, ions and biomolecules on the fluorescence properties of 4b

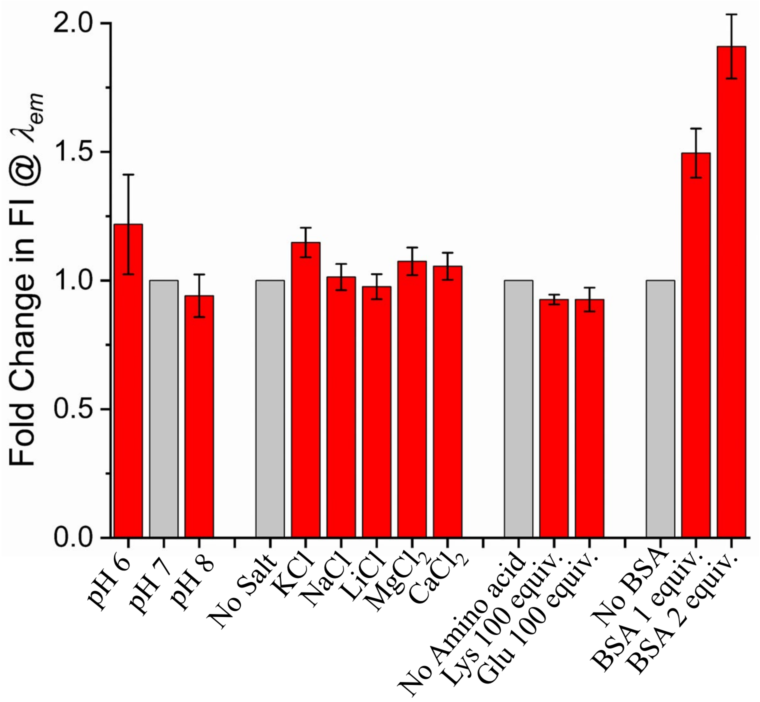

Figure S15. Emission studies of **4b** (c_{4b} = 3 µM) at different pHs using sodium cacodylate buffer ($c_{\text{Na-cacodylate buffer}}$ = 100 mM). Emission studies of **4b** (c_{4b} = 3 µM) in the absence and presence of different salts (c_{salt} = 100 mM, c_{TRIS} = 50 mM, pH: 7.4). Emission studies of **4b** (c_{4b} = 3 µM) in the absence and presence of lysine, glutamic acid and BSA ($c_{\text{amino acid}}$ = 300 µM, c_{BSA} = 3 or 6 µM, c_{TRIS} = 50 mM, pH: 7.4). λ_{exc} = 513 nm. Data were normalized based on the experimental conditions reported in the gray bars.

UV/Vis titration studies of 4b and parallel G4s along with the 1:1 or 1:2 binding models

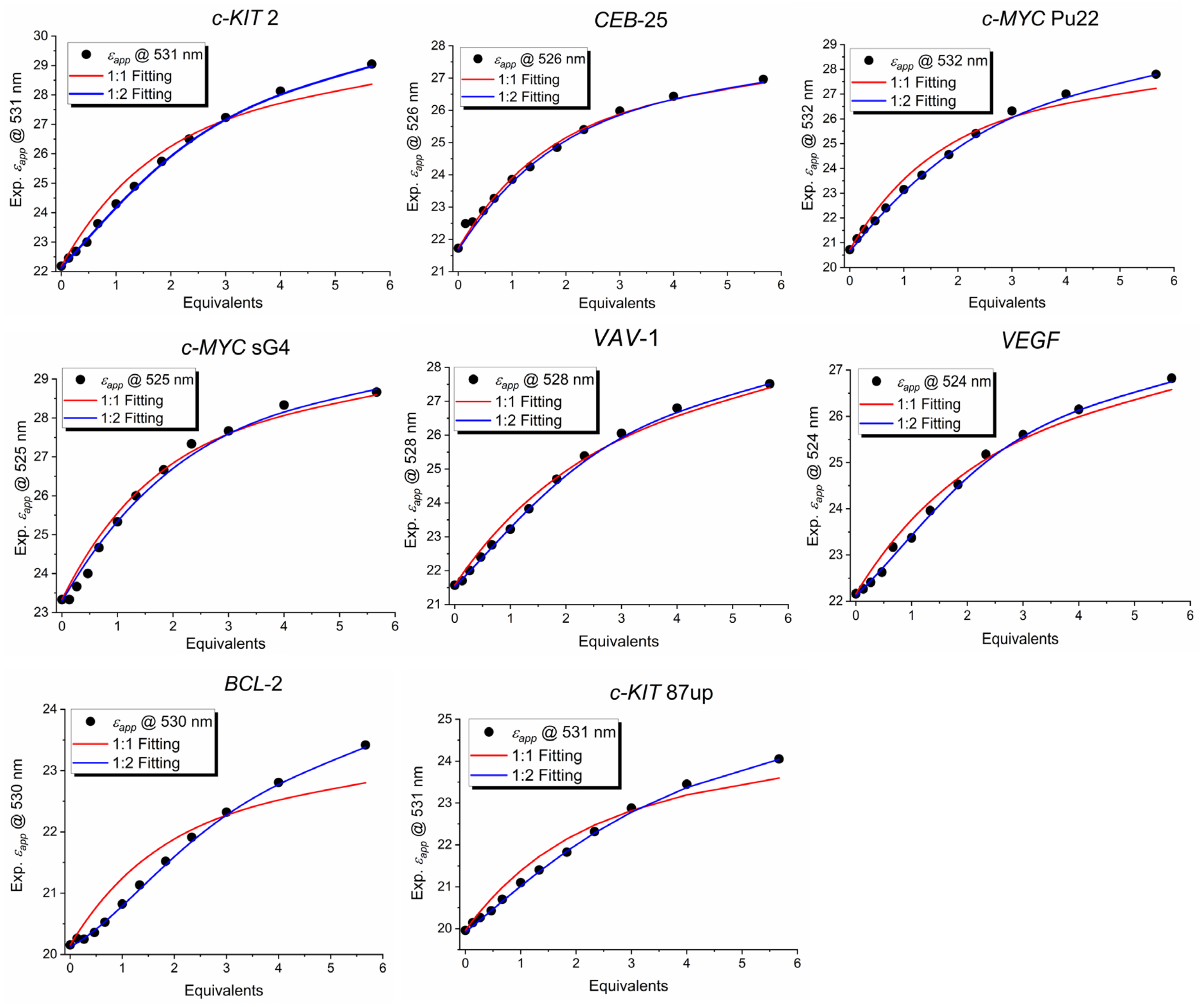

Figure S16. Spectrometric titrations of **4b** complexed with parallel G4s (c_{4b} = 3µM, $c_{parallel\ G4s}$ = 0 to 17, c_{KCl} = 100 mM, c_{TRIS} = 50 mM, pH: 7.4). The red and blue lines result from the fitting procedure using a 1:1 or 1:2 binding model. The fitting output are summarized in Table S2.

Global fitting data analysis of 4b in the presence of parallel G4s

1:1 binding model **1:2 binding model**

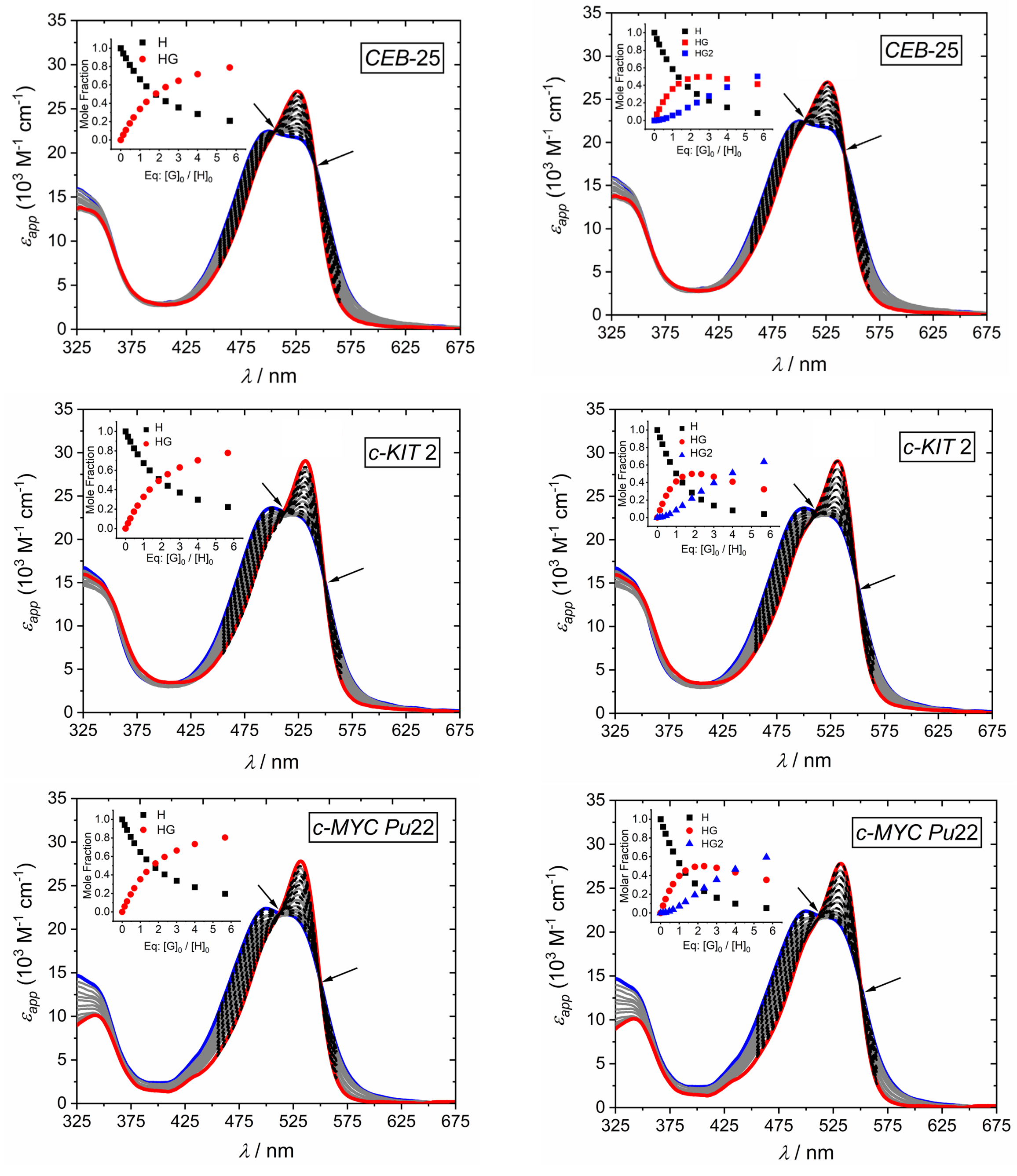

Figure S17. UV/Vis absorption spectra of **4b** in the absence (blue line) and presence (gray and red lines) of increasing concentrations of parallel G4s (c_{4b} = 3 µM, $c_{parallel\ G4s}$ = 0 to 17 µM, c_{KCl} = 100 mM, c_{TRIS} = 10 mM, pH: 7.4). Superimposed black dashed line results from a global fitting with either a 1:1 or 1:2 binding model. The solid arrows indicate the appearance of isosbestic points. The insets show the speciation analysis for the titration of **4b** with parallel G4s. $[G]_0$ = free G4 concentration, $[H]_0$ = free **4b** concentration. H, HG and HG2 are the components present in the system and indicate: **4b**, **4b**-(*c-KIT* 2) 1:1 adduct and **4b**-(*c-KIT* 2)2 1:2 adduct, respectively.

Tangent method plot

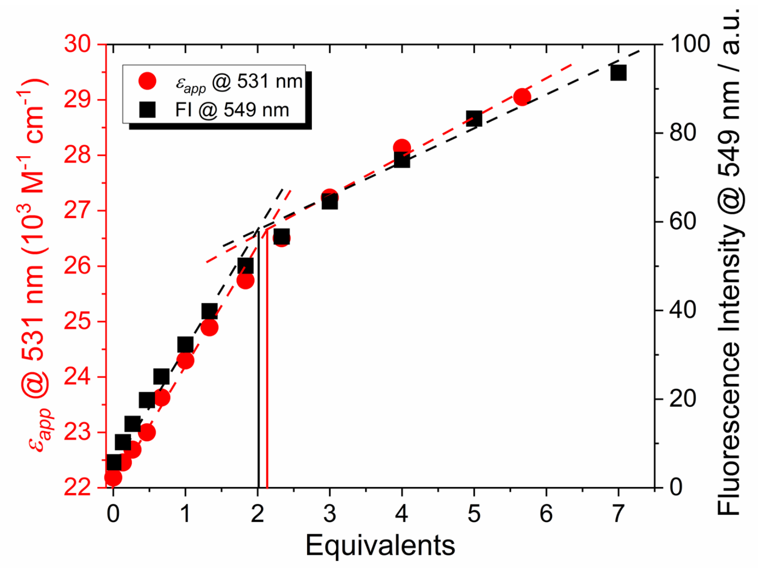

Figure S18. Tangent method analysis for **4b**-*c-KIT* 2 system supporting the 1:2 binding stoichiometry.

Fluorescence kinetic traces of 4b bound to *c-KIT* 2

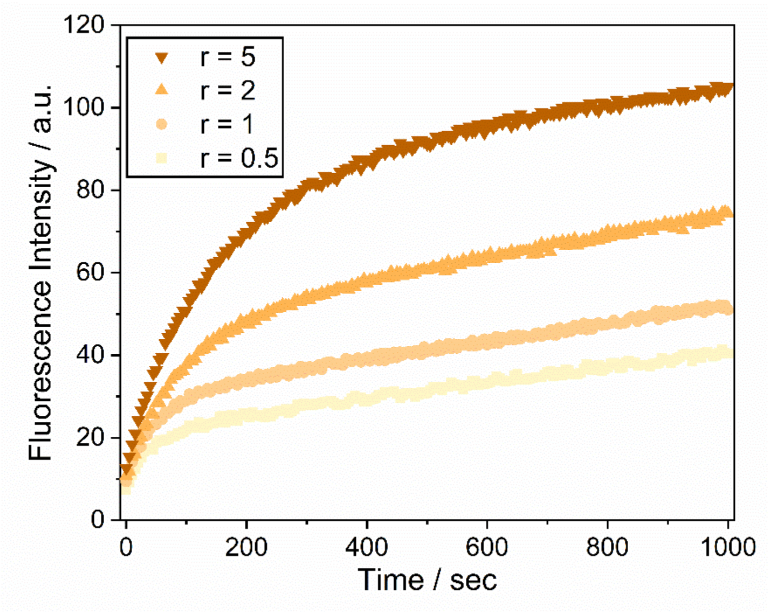

Figure S19. Time-dependent fluorescent spectral profile of **4b** complexed with *c-KIT* 2 at different *c-KIT* 2/**4b** molar ratios (r).

CD spectra

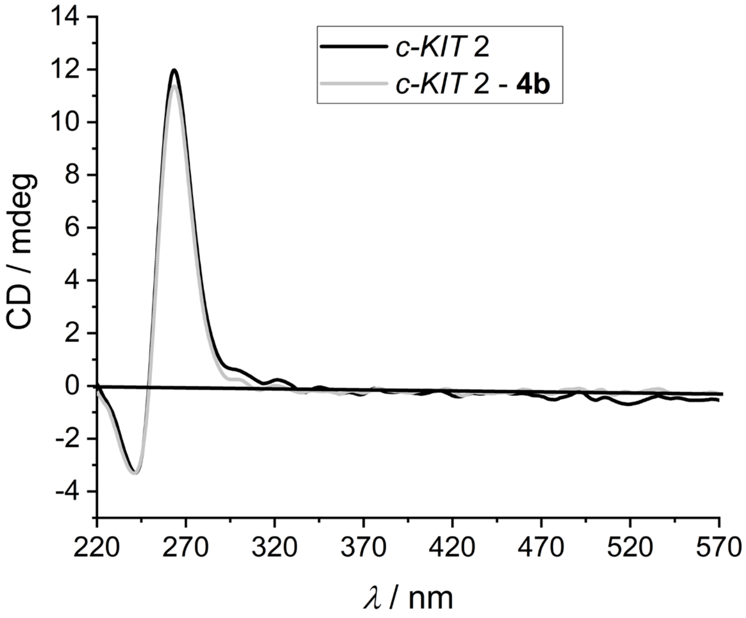

Figure S20. CD spectra of *c-KIT* 2 and *c-KIT* 2-**4b** complex ($c_{\mathbf{4b}}$ = 2 µM, $c_{c\text{-}KIT\,2}$ = 1 µM, c_{KCl} = 100 mM, c_{TRIS} = 10 mM, pH: 7.4).

Fluorescence displacement assay

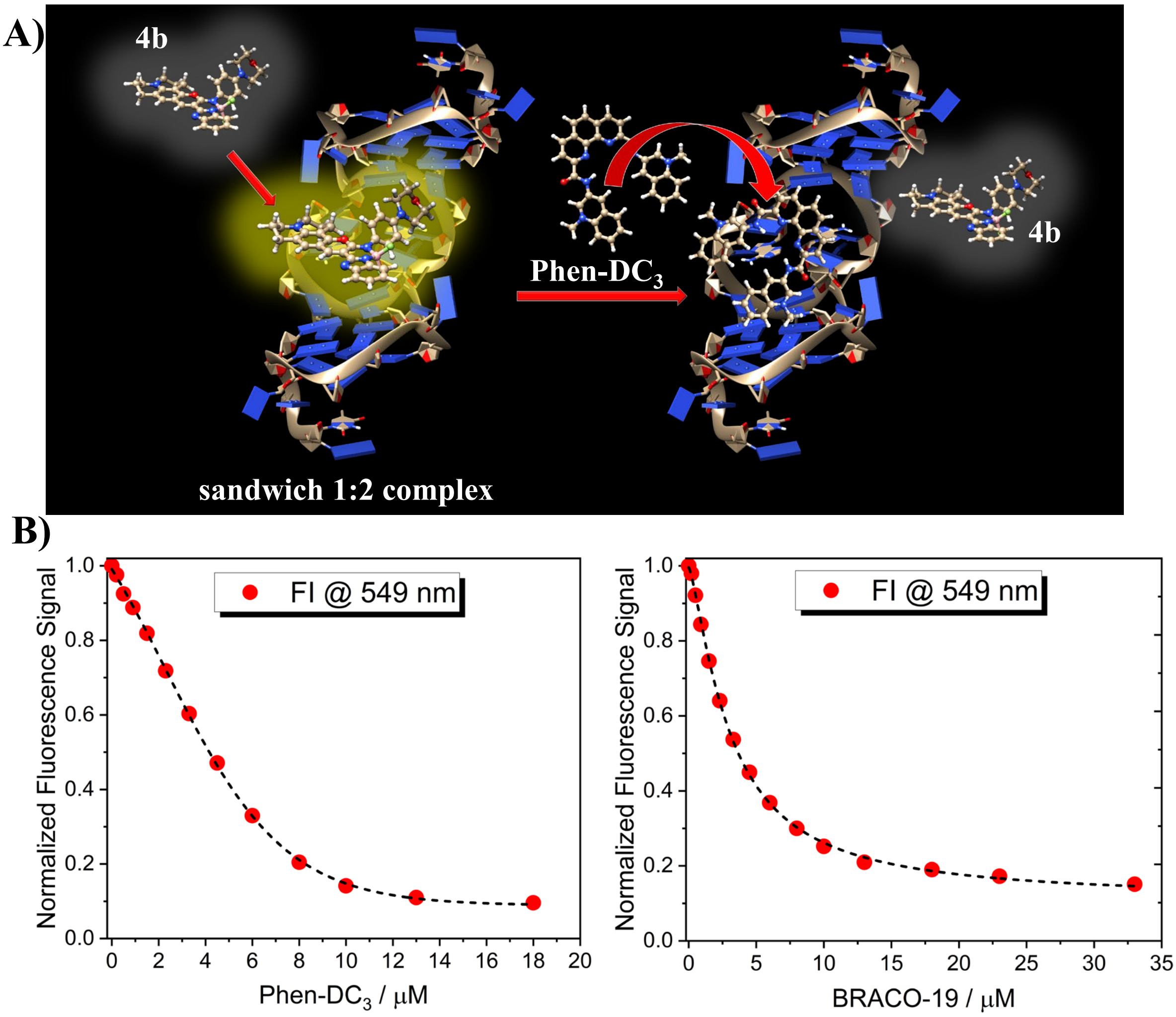

Figure S21. A) Schematic illustration of the fluorescence displacement assay performed by using the G4 end-stackers Phen-DC3 and BRACO-19. **4b** is non-emissive in its free state. Complexation with parallel G4s through a 1:2 sandwich-like complex results in the enhancement of **4b** fluorescence intensity. Replacement of **4b** from the G4 template by Phen-DC3 or BRACO-19 caused the fluorescence quenching of **4b**. B) Fluorescence emission changes on the **4b**-*c-KIT 2* system upon increasing concentration of Phen-DC3 or BRACO-19 (c_{4b} = 3 µM, $c_{c\text{-}KIT\,2}$ = 6 µM, $c_{Phen\text{-}DC3}$ = 0 to 18 µM, $c_{BRACO\text{-}19}$ = 0 to 33 µM, c_{KCl} = 100 mM, c_{TRIS} = 10 mM, pH: 7.4, λ_{exc} = 513 nm).

^{1}H NMR spectra of *c-MYC* Pu22 G4 at various DMSO-d_6 percentage

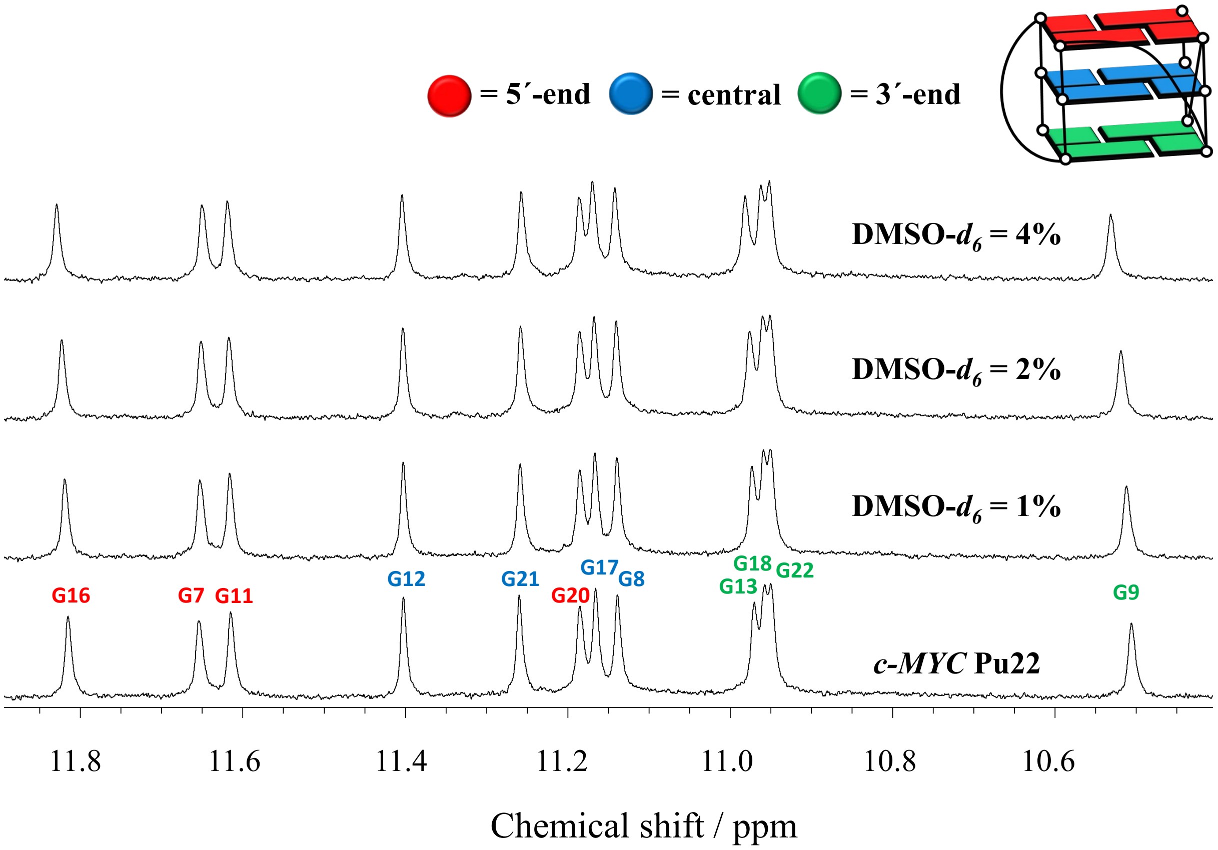

Figure S22. ^{1}H NMR spectra showing the evolution of the imino-region of *c-MYC* Pu22 at different percentage of DMSO-d_6.

Supplementary Table 1. List of the biologically relevant natural and synthetic oligonucleotides used for biophysical characterization.

Name	Sequence [a]	Length / bp	Absorptivity [b] / M^{-1} cm^{-1}	GC / %	Topology
c-MYC Pu22	TGAGGGTGGGTAGGGTGGGTAA	22	228700	59.1	parallel
c-MYC sG4	GGGTGGGTAGGGTGGG	16	162700	75	parallel
VAV-1	GGGCAGGGAGGGAACTGGG	19	194700	73.7	parallel
VEGF	GGGAGGGTTGGGGTGGG	17	171400	76.5	parallel
c-KIT 2	CCCGGGCGGGCGCGAGGGAGGGGAGG	26	253400	88.5	parallel
c-KIT 87up	AGGGAGGGCGCTGGGAGGAGGG	22	226700	77.3	parallel
CEB-25	AGGGTGGGTGTAAGTGTGGGTGGGT	25	253100	60.0	parallel
BCL-2	GGGCGCGGGAGGGAATTGGCGGGG	24	237400	79.2	parallel
Tel-22	AGGGTTAGGGTTAGGGTTAGGG	22	228500	54.5	hybrid
Bom17	GGTTAGGTTAGGTTAGG	17	174600	47.1	antiparallel
TBA	GGTTGGTGTGGTTGG	15	143300	60	antiparallel
ss-DNA	TTACCCACCCTACCCACCCTCA	22	191500	59.1	ss-DNA
ds-DNA	CAATCGGATCGAATTCGATCCGATTG	26	253200	46.2	ds-DNA [c]

[a] Conventional 5′ to 3′ direction. [b] Molar extinction coefficient calculated by using oligo analyzer on the IDT web site. [c] self-complementary DNA duplex.

Supplementary Table 2. Binding models and associated fitting output.

Technique	System	K / 10^5 M^{-1}	Error %	Covariance	K_{12} / 10^5 M^{-1}	Error %	Covariance
UV/Vis	*c-KIT* 2	2.4	1.728	0.011242	6.5	1.210	0.002963
Emission	*c-KIT* 2	1.1	0.166	0.000259	1.8	0.221	0.000322
UV/Vis	*c-MYC* Pu22	2.8	1.756	0.010102	5.5	1.645	0.005379
Emission	*c-MYC* Pu22	1.7	0.181	0.000233	2.4	0.215	0.000222
UV/Vis	*CEB*-25	2.6	0.896	0.003093	3.8	1.019	0.002469
Emission	*CEB*-25	0.9	0.213	0.000489	1.5	0.258	0.000524
UV/Vis	*c-MYC* sG4	2.5	1.657	0.011008	1.1	1.042	0.005357
Emission	*c-MYC* sG4	1.9	0.331	0.000680	2.8	0.408	0.000686
UV/Vis	*BCL*-2	2.3	2.348	0.026111	5.9	1.821	0.009364
Emission	*BCL*-2	0.5	0.212	0.000706	2.0	0.229	0.000503
UV/Vis	*VAV*-1	1.3	1.049	0.006427	7.0	1.138	0.003196
Emission	*VAV*-1	0.6	0.205	0.000593	2.1	0.222	0.000427
UV/Vis	*VEGF*	1.5	1.305	0.010498	9.1	1.457	0.004654
Emission	*VEGF*	1.0	0.241	0.000591	0.9	0.193	0.000318
UV/Vis	*c-KIT* 87up	1.9	1.490	0.011189	4.3	1.271	0.005078
Emission	*c-KIT* 87up	0.3	0.233	0.001091	1.3	0.268	0.000942

Supplementary Table 3. List of the biologically relevant mtDNA G4s used for biophysical characterization. Their folding pattern has been previously characterized.[3]

Name	Sequence [a]	Length / bp	Absorptivity [b] / M^{-1} cm^{-1}	GC / %	Topology
Mito 112	GGGGTTGAGGGATAGGAGGAGAATGGGGG	29	308200	62.1	parallel
Mito 126	GGGAGGTTGAAGTGAGAGGTATGGT	25	262300	52.0	parallel
Mito 143	GTGGGGTGAAAGAGTATGATGGGGTGGTGG	30	310400	56.7	parallel
Mito 55	AGGGCGATGAGTGTGGGGAGGAATGGGGTGGGT	33	339100	63.6	Parallel
Mito 3	CGGGGGGAGGGGGGGTTTGGTGGA	24	240100	75.0	parallel
Mito 95	AGGAGGGGGGTTGTTAGGGGGTCGGAGGAAAAG GTTGGGGA	41	427500	61.0	parallel
Mito 124	GGGGGGTGGAAGCGGATGAGTAAGAAG	27	287800	59.3	parallel
Mito 12	TGGGGGTGTGGCTAGGCTAAGCG	23	222300	65.2	hybrid
Mito 117	AGGGAGAGCTGGGTTGTTTGGGTTGTGG	28	274100	57.1	hybrid
Mito 30	CGGCAAGGTCGAAGGGGGTTCGGTTGGT	28	272300	64.3	antiparallel
Mito 119	GGCCTAGTAGTGGGGTGAGGCTTGG	25	243500	64.0	antiparallel
Mito 78	ATGGGTTTGGTGAGGGAGGTAGGTGGT	27	274400	55.6	antiparallel
Mito 0.5-21	GGGGATGGCCATGGCTAGG	19	186500	68.4	antiparallel
Mito 0.5-29	GGTTAGGTAGTTGAGGTCTAGGG	23	234000	52.2	antiparallel
Mito 0.5-2	GGTTAGGCTGGTGTTAGGG	19	186800	57.9	antiparallel

[a] Conventional 5′ to 3′ direction. [b] Molar extinction coefficient calculated by using oligo analyzer on the IDT web site.

References

(1) Thordarson, P. Determining association constants from titration experiments in supramolecular chemistry. *Chem. Soc. Rev.* **2011**, *40* (3), 1305-1323.
(2) von Krbek, L. K. S.; Schalley, C. A.; Thordarson, P. Assessing cooperativity in supramolecular systems. *Chem. Soc. Rev.* **2017**, *46* (9), 2622-2637.
(3) Bedrat, A.; Lacroix, L.; Mergny, J.-L. Re-evaluation of G-quadruplex propensity with G4Hunter. *Nucleic Acids Res.* **2016**, *44* (4), 1746-1759.
